# Carabid beetle traits tell tales of urban filtering: shifts in activity, flight behaviour, reproduction, and overwintering

**DOI:** 10.1007/s11252-026-02128-8

**Published:** 2026-09-15

**Authors:** Wouter B. van der Vegt, Jacintha Ellers, Nienke A. Schijfsma, Olaf T. van der Woude, Joscha Beninde, Matty P. Berg

**Affiliations:** 1 Section Ecology & Evolution, A-LIFE, Vrije Universiteit Amsterdam, De Boelelaan 1108, 1081 HZ Amsterdam, The Netherlands; 2 Conservation Ecology Group, GELIFES, University of Groningen, Groningen, The Netherlands

**Keywords:** City, Community assembly, Functional diversity, Green space, Ground beetle, Urbanisation

## Abstract

Urban environments are characterised by habitat fragmentation, sensory and chemical pollution, and altered climatic conditions. Species may either adapt to these stressors or already exhibit pre-adaptive traits. Traits mediate how species respond to cities and influence their persistence. Cities often host unique species assemblages compared to rural sites, and traits could be instrumental in understanding these community compositional changes. An array of carabid beetle species are commonly found in urban and rural environments and their ecology and traits are well known. Here, we study the trait composition and functional diversity in carabid beetle communities in response to urbanisation. We sampled carabid beetles with pitfall traps across four replicated urbanised landscapes in the Netherlands, in city centres and peaty rural pastures. We found a consistent shift in species composition between rural and urban environments, more species in rural areas but no difference in abundance. In contrast to rural communities, urban communities were characterised by later-reproducing, less frequently flying, more nocturnal and eurytopic species that more frequently overwinter as larvae. Also, we found lower functional richness and higher functional divergence in urban communities, but no difference in functional evenness and functional dispersion compared to rural sites. Our results contribute novel trait-urbanisation associations compared to earlier studies, which may be due to different geographic regions and studied urbanised landscapes and their associated differences in environment and habitat. Overall, these results hint that environmental filtering shapes urban communities. Future studies could investigate how habitat-specific characteristics influence trait composition.

**Supplementary Information:**

The online version contains supplementary material available at https://doi.org/10.1007/s11252-026-02128-8.

## Introduction

Urbanisation has been expanding rapidly in recent decades, leading to vast land use changes worldwide. Urban environments are characterised by a high degree of impervious surface with a mosaic of isolated and fragmented green spaces that often differ in environmental conditions compared to rural sites (Perry et al. 2020). The distinct combination of abiotic and biotic landscape features (Santangelo et al. 2022), such as high pollution (e.g., chemicals, noise, and light), increased temperatures, and altered resource availability, affects evolutionary processes and dispersal (Johnson and Munshi-South 2017; Piano et al. 2023), and shapes urban species distribution and community composition (Groffman et al. 2014; Aronson et al. 2014; Beninde et al. 2015; Ooms et al. 2020). To cope with the altered conditions in urban environments, species may need to evolve adaptive traits in situ. Evolutionary adaptation to urban environments can lead to intraspecific trait divergence and distinct urban populations, as seen in songs of great tits, skull morphology of red foxes and tibiae length of carabid beetles (Mockford and Marshall 2009; Parsons et al. 2020; Magura et al. 2025). Alternatively, species that possess traits beneficial for city life may occur in urban environments as a result of environmental filtering. Environmental filtering, i.e. abiotic factors preventing the establishment or persistence of species in a particular location (Kraft et al. 2015), may be more pronounced in urban than in rural environments because urban environments are typically associated with higher temperatures, higher drought stress, increased habitat fragmentation and more impervious surfaces (Grimm et al. 2008; Youngsteadt et al. 2015; Fournier et al. 2020). This may impose stronger selection on species’ traits that allow species to tolerate these urban conditions. For example, generalist species may be better able to exploit opportunities in urban environments, and species with traits showing high phenotypical plasticity (Jones and Leather 2012; Géron et al. 2024) may be able to adjust their traits to urban conditions. This environmental filtering results in contrasting species assemblages in urban and the surrounding non-urban habitats (Niemelä and Kotze 2009; Bang and Faeth 2011; Bogyó et al. 2015), typically leading to a lower species richness (Faeth et al. 2011; Fenoglio et al. 2020; Szabó et al. 2023). Here, we focus on the traits relevant to pass the urban environmental filter to gain a better understanding of what enables species to occur in these human-dominated environments.

Trait ecology can be useful to understand compositional differences between species communities in urban and non-urban environments (McGill et al. 2006), as differences in traits aid or constrain species acting in urban environments (Dahirel et al. 2017). Traits describe how a species lives in and interacts with its environment, for example: the level of eurytopy, fly frequency and nocturnality. Given the unique abiotic and biotic characteristics of urban environments, specific trait combinations are expected to become dominant in urban communities. For instance, urban arthropods have been shown to be more drought-tolerant, better dispersers, and more frequently have a generalist diet and/or niche breadth than rural ones (McIntyre et al. 2001; McKinney 2008; Menke et al. 2011; Magura et al. 2018). Body size is also a trait that has been shown to respond to urbanisation, but patterns differ sharply between taxa (Merckx et al. 2018). Although these studies provide valuable insights into the effects of single traits, studies exploring multiple traits simultaneously are currently scarce, but necessary to identify and distinguish the main drivers of compositional differences between urban and rural environments (Fournier et al. 2020; Pernat et al. 2024).

To address this question, we use carabid beetles (Coleoptera: Carabidae) as a model system. They are common in both urban and rural environments, their ecology is well known, and they react to environmental changes, such as human disturbance or urbanisation (Lövei and Sunderland 1996; Turin 2000; Rainio and Niemelä, 2003; Koivula, 2011). In addition, trait data for many species are available, which makes carabid beetles particularly well-suited to explore species compositions across urban to rural environments. Studies report urban carabid beetles to be typically smaller (Hahs et al. 2023; Kotze et al. 2024; Merckx et al. 2018; but see Magura et al. 2020); more often generalists (Fournier et al. 2020; Hahs et al. 2023; Kotze et al. 2024); and better dispersers compared to rural species (Niemelä and Kotze 2009; Piano et al. 2017; Hahs et al. 2023; Kotze et al. 2024). Also, species in relatively warmer regions (e.g., Central Europe) are more often nocturnal than conspecifics from colder places (Arctic) (Lövei and Sunderland 1996), suggesting that temperature influences diel activity. This relationship may also act on a more local scale when comparing urban with surrounding rural environments. The abovementioned traits could be imperative for carabid beetles to persist within and navigate the more isolated and warmer habitat patches of urban environments (e.g., by avoiding heat and traffic). Moreover, the studies of Kotze et al. (2024) and Hahs et al. (2023) suggest using multiple traits is valuable for understanding compositional changes across urban–rural environments. Hahs et al. (2023) showed in their functional diversity analyses that functional richness generally increases with urbanisation, while functional evenness and functional dispersion decrease. In our study, we include three additional traits; (1) nocturnality, (2) timing of reproduction and (3) number of active months compared to Hahs et al. (2023) and Kotze et al. (2024), to assess whether urban–rural compositional trait differences are found consistently and whether these additional traits contribute in explaining compositional differences and functional diversity patterns.

To answer these questions, we collected carabid beetles in replicated urban (parks and courtyards) and adjacent peaty rural (grassy road verges and pastures) sites from four cities in the Netherlands. Recent studies emphasise the importance of including multiple cities when making assessments about trait compositions (Evans et al. 2009; Walker 2024). Further, we compiled an extensive country-specific trait dataset, containing values for the average body length, fly frequency, level of eurytopy, moisture preference, nocturnality, number of active months, overwintering stage and timing of reproduction from specialised Dutch literature. We hypothesise that: (i) the community composition differs between urban and rural environments, with a higher species richness in the rural environment compared to urban environments; (ii) the trait composition differs between both environments, and we expect urban communities to be associated with higher fly frequency, higher level of eurytopy, and more nocturnality; and (iii) the functional diversity in urban environments is lower than in rural environments. We expect strong environmental filtering in cities where species with high levels of eurytopy and dispersal capacities persist, resulting in low trait diversity.

## Materials and methods

### Study sites

**Fig. 1:**
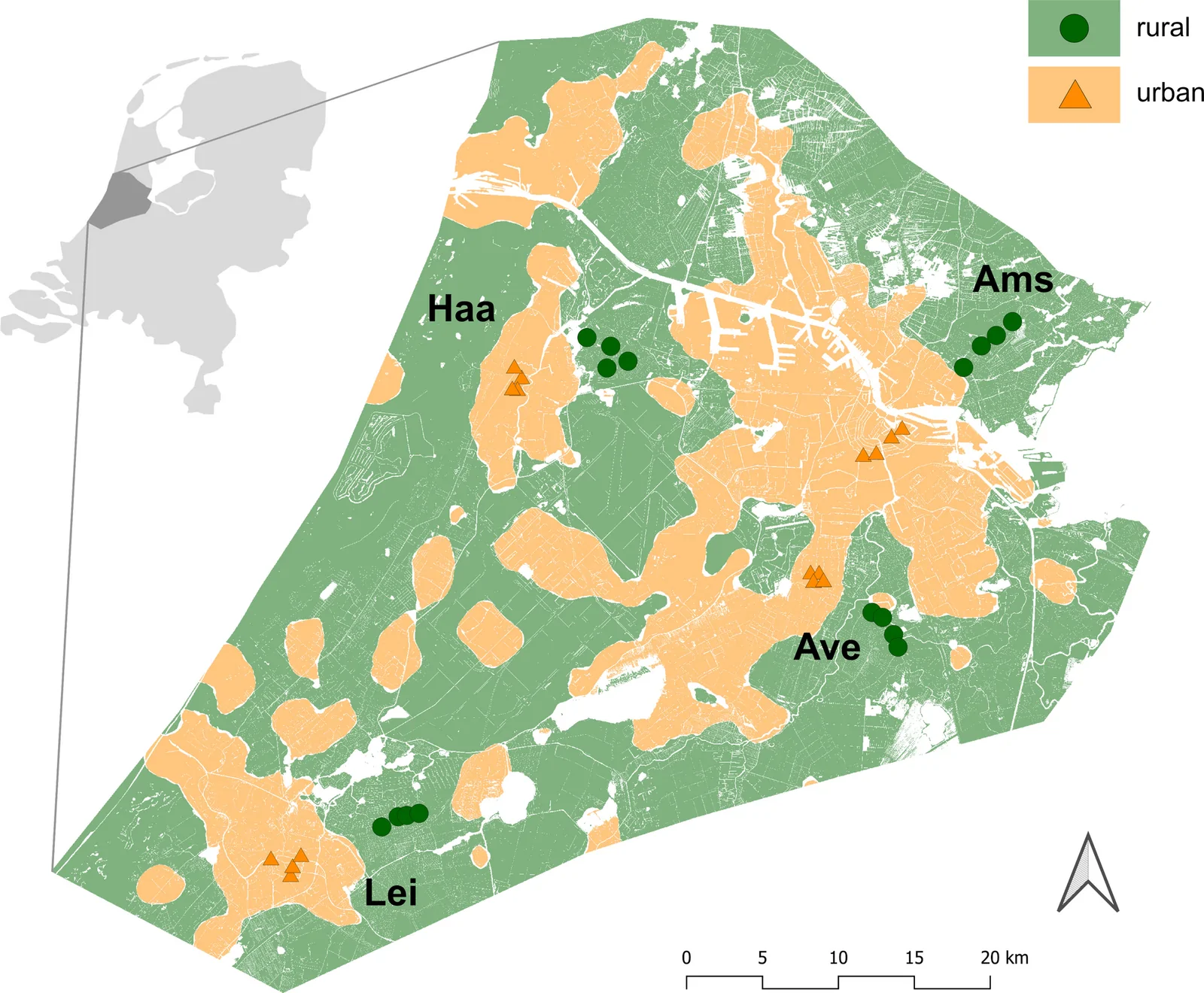
Study sites (symbols) across four Dutch cities with urban and rural categorisation. Orange shows urban environments (proportion impervious surface cover ~ 0.58), green rural (proportion impervious surface cover = 0.11), and white water surfaces across the entire area. Orange triangles resemble urban sites and green circles rural sites. Abbreviations represent the cities (Ams = Amsterdam, Ave = Amstelveen, Haa = Haarlem, and Lei = Leiden)

To assess carabid beetle species and trait compositions sampling was replicated in four cities: Amstelveen (Lat. 52.303580, Long. 4.860767), Amsterdam (Lat. 52.377956, Long. 4.897070), Haarlem (Lat. 52.387386, Long. 4.646219), and Leiden (Lat. 52.160114, Long. 4.497010) (Fig. 1, Online Resource Table S1). All cities had similar average annual temperatures and precipitation (Online Resource Table S2). Each city was sampled at four urban and four adjacent rural sites, with at least 350 m distance between all sites. Urban and rural areas were distinguished using impervious surface raster data (Ren et al. 2024), while keeping vegetation structure as similar as possible. Clustering analyses were performed twice, based on the mean proportion of impervious surface cover within 1,250 m and 1,500 m of each raster cell. To increase environmental similarity within urban and rural sampling sites, only those cells belonging to the same cluster at both distances were retained and deemed suitable sampling locations (Fig. 1). Urban clusters had a mean proportion of 0.58 and 0.57 impervious surface cover at 1,250 m and 1,500 m, respectively, while rural clusters had mean proportions of 0.11 at both distances. The between-cluster sum of squares accounted for 79.7% and 79.2% of the total variation, respectively. Sampling sites in urban areas represented parks, courtyards, and green corridors. All urban sites were publicly accessible and were managed. In rural areas, sampling was done on peat soil and was mostly carried out along grassy road verges or within pastures. They were also managed, and as a result, all sample sites had some level of management in common, but the type of management differed between urban (hoeing) and rural (mowing). Sampling sites (both urban and rural) were located near shrubs, bushes, and trees where possible. In rural sites, this was not always possible due to a lack of trees or shrubs.. In the absence of shrubby vegetation at rural sites, pitfalls were placed in tall grass vegetation. Hence, for all traps, exposure to direct sunlight was prevented. Vegetation cover was higher in rural areas (mean: 84.6%) than in urban areas (60.5%) and vegetation height was also, on average, higher in rural areas (52.5 cm) than in urban areas (41.3 cm).

### Sampling process

Pitfall traps (*n* = 5 per site; depth = 12 cm; diameter = 11 cm) were placed at least 5 m apart from each other at each site. They were filled to a third with a saturated saline solution (cooking salt and tap water). To protect pitfalls from direct sunlight, precipitation and debris, polyvinyl chloride (PVC) roofs (17 × 17 cm) were placed two centimetres above the traps with pegs. The pegs were placed in the soil and the roof was kept at a consistent height by rubber O-rings around the pegs just beneath the roof (Online Resource Fig S1). Traps were fortnightly emptied and refilled with new fixation fluid (starting from 17 April 2023 till 7 July 2023, for Haarlem an additional round was done from 16 August 2023 till 30 August 2023). Carabid beetles were separated from the bycatch and stored in 70% ethanol per trap in the laboratory. All specimens were identified to species level with Dutch identification keys (Boeken et al. 2002; Muilwijk et al. 2015). We made a reference collection for each species to facilitate specimen identification. Difficult species identifications were validated by carabid beetle experts M. P. Berg or M. Boeken.

### Selection of traits

Trait information and values specifically for Dutch carabid beetle species were collected from Turin (2000), Boeken et al. (2002) and Muilwijk et al. (2015) and compiled into a trait database. From the available traits, only those that were assumed to have relevance to species performance in either the urban or rural environment were selected (Table 1). For traits with uncertain values (two for nocturnality), we first relied on experts' descriptions of their likely values and, where necessary, inferred trait values from congeneric species with known values. Overall, only five trait data values were missing (0.7%), which we imputed from congeneric species via Multivariate Imputation by Chained Equations (MICE) v3.18.0 with predictive mean matching and ten imputations (Buuren and Groothuis-Oudshoorn 2011) (Online Resource Table S3). It is important to note that we used fixed species-specific trait values, therefore intraspecific trait variation and possible local adaptation in trait values to urbanisation were ignored.

**Table 1:** Summary of the traits, their abbreviation as used in analyses, their units, and description and origin of the literature source for trait information used in our study

Traits	Traits abbreviated	Data type and unit/scale	Description	References
Average body length	avg_length	Continuous (mm)	The average body size is calculated by the mean of the minimum and maximum length of a species in mm	Boeken et al. 2002; Muilwijk et al. (2015)
Habitat moisture preference	moisture_pref	Ordinal (1–5)	Moisture preference for carabid beetles (1 = dry; 2 = dry/moist; 3 = intermediate moisture preference; 4 = moist/wet; 5 = wet)	Turin (2000)
Level of eurytopy	eurytopy	Ordinal (1–10)	Depicts how specialised (= the level of eurytopy) a species is regarding different habitats (1 = extremely stenotopic, found in a single biotope; 2 = stenotopic; 3 = fairly stenotopic; 4 = moderately stenotopic; 5 = intermediate; 6 = moderately eurytopic; 7 = fairly eurytopic; 8 = eurytopic; 9 = very eurytopic; 10 = extremely eurytopic, found in all biotopes)	Boeken et al. (2002); Turin (2000)
Fly frequency	fly_freq	Ordinal (1 or 2)	How often a species has been observed flying (1 = never or rarely; 2 = frequently)	Turin (2000)
Timing of reproduction	reproduction	Ordinal (1–12)	The moment in the year that a species starts reproducing (1 = winter, 2 = winter-early spring; 3 = early spring; 4 = spring, 5 = primarily spring but also autumn; 5.5 = spring–summer; 6 = early summer; 7 = summer; 8 = late-summer; 8.5 = summer-autumn; 9 = primarily autumn but also spring; 10 = autumn; 11 = late autumn; 12 = early winter). Values are derived from the associated months and decimal values indicate the mean of corresponding period values when appropriate	Turin (2000)
Overwintering stage	winter_stage	Ordinal (1–3)	The overwintering stage of a species (1 = larvae; 2 = larvae or adult; 3 = adult)	Turin (2000)
Number of active months per year	months_active	Discrete (months)	The number of months that a species is active in a year. Calculated by subtracting the first month active from the last month active	Muilwijk et al. (2015)
Nocturnality	nocturnality	Ordinal (1–5)	Period of the day in which a species is primarily active (1 = night; 2 = primarily night; 3 = day and night; 4 = primarily day; 5 = day)	Turin (2000)

In total, we included eight traits (Table 1): (1) Body size (average length) is a proxy for multiple other traits (e.g., thermal and desiccation tolerance, and fecundity) (Ellers and Jervis 2003; Chown and Nicolson 2004) and may differ between urban and rural habitats as a response to environmental changes. (2) Moisture preference strongly shapes species-specific distributions (Rainio and Niemelä 2003; Niemelä et al. 2007) and may act as an environmental filter in cities, where soils are generally drier (Savva et al. 2010). (3) The level of eurytopy indicates how generalist or specialist a species is, and thus indirectly the ability to persist in disturbed habitats such as cities. (4) Fly frequency (i.e., how often a species has been observed flying) reflects the dispersal capacity of carabid beetles better than wing morphology. Although some species are macropterous, which usually indicates that a specimen can fly, they have not necessarily been frequently observed flying (e.g., *Amara ovata, Agonum viduum, Badister bullatus, Nebria brevicollis)* or not at all (e.g., *Badister lacertosus, B. sodalis, Harpalus anxius, Leistus fulvibarbis*) (Turin 2000). In a patchy urban environment, flying may be essential for navigating to different patches while avoiding dangerous traffic. (5) The timing of reproduction may be shifted by higher temperatures caused by the urban heat island effect, whereby resource availability or biological processes are altered. (6) The overwintering stage in urban environments may consist of a higher proportion of larvae than adults compared to rural environments. This is due to higher temperatures in cities (Smith et al., 2011), which could lead to extended reproductive periods. (7) The number of months a species is active may be higher in urban habitats where temperatures are higher. Hereby, it advances the moment they become active in the spring and allows them to remain active for longer in the autumn, indicating how long they can profit from resources in the area. Species that are already known to be active for an above-average number of months could benefit more from this than those with a limited number of months of activity. (8) Lastly, the nocturnality can be higher in urban settings to avoid traffic and higher temperatures during the day or as a consequence of artificial light at night.

### Data analyses

Sample-size-based rarefaction and extrapolation curves and sample coverage were calculated via the iNEXT package v3.0.2 (Online Resource Table S4). Sites with coverage < 0.75 were removed from all analyses (i.e., one rural site from Amstelveen and one rural site from Leiden). Additional rarefaction curves were made for all sites combined per city (Online Resource Fig S2A), urban sites combined per city (Online Resource Fig S2B), and rural sites combined per city (Online Resource Fig S2C). To investigate if abundance and species richness differed between habitats, the total abundance and number of species for each site were calculated. Abundance and species richness were analysed with generalised linear mixed models with habitat as a fixed effect and city as a random effect with a negative binomial distribution using “glmmTMB” v1.1.11 (Brooks et al. 2017).

To assess whether urban species compositions differed from rural compositions, we performed a non-metric multidimensional scaling (NMDS) ordination. The total number of individuals per site was determined by compiling all specimens from the traps from all collection moments. First, data were square root transformed, followed by Wisconsin double standardisation to reduce the influence of the most dominant species, while increasing the rarer species and to make relative species abundances per site. This transformation and standardisation frequently improve the quality of ordinations, although conditions on when to use these are arbitrary (Oksanen et al. 2025) Then, Bray–Curtis distances were calculated with 999 permutations and utilised for NMDS plots and statistical testing for differences in species composition with the adonis2 test from the “vegan” package v2.6-10 (Oksanen et al. 2025), with habitat as a fixed effect and city as a random effect.

Differences in trait compositions of urban and rural communities were assessed by first calculating the community weighted mean (CWM) value of all traits per site. CWMs are commonly used to assess shifts in communities mediated by environmental disturbance and can be used to explain ecosystem functioning (Violle et al. 2007; Gaüzère et al. 2019). Then, by using these CWMs, a PCoA with Gower’s distance and Lingoes correction was done to assess which traits are featured in urban and which in rural habitats with “ape” v5.8.1 (Paradis and Schliep 2019). The difference in trait composition was assessed with the adonis2 test. We also evaluated if CWM trait values differed per location and habitat with estimated marginal means comparisons (if linear model assumptions were not violated) or Dunn tests (if violated) (Online Resource Fig S3, Table S5). The importance of traits in shaping the ordination has been analysed using the r^2^ values from the envfit function in the “vegan” package.

Functional diversity of urban and rural communities was assessed with functional richness (FRic), functional divergence (FDiv), functional evenness (FEve), and functional dispersion (FDis) based on Gower’s distance with “mFD” v1.0.7 (Magneville et al. 2022). FRic describes the extent of trait diversity represented by the collected specimens; FDiv the degree to which species abundances are concentrated toward the extremes of the trait space; FEve to what extent the specimens’ abundances are evenly spread across the trait space they occupy; FDis the abundance-weighted mean distance of individual species to the centroid of all species in the trait space (Laliberté and Legendre 2010; de Bello et al. 2021). This was followed by linear mixed models (for FRic) and generalised linear mixed models with, depending on model diagnostics, a beta distribution (FEve, FDiv) and Gaussian (FDis) distribution, with city as a random effect. Generalised linear mixed model assumptions were checked with “DHARMa” v0.4.7 (Hartig et al. 2024). All analyses were conducted in R using v4.5.2 (R Core Team 2025).

## Results

### Abundance, species richness and species composition

**Fig. 2:**
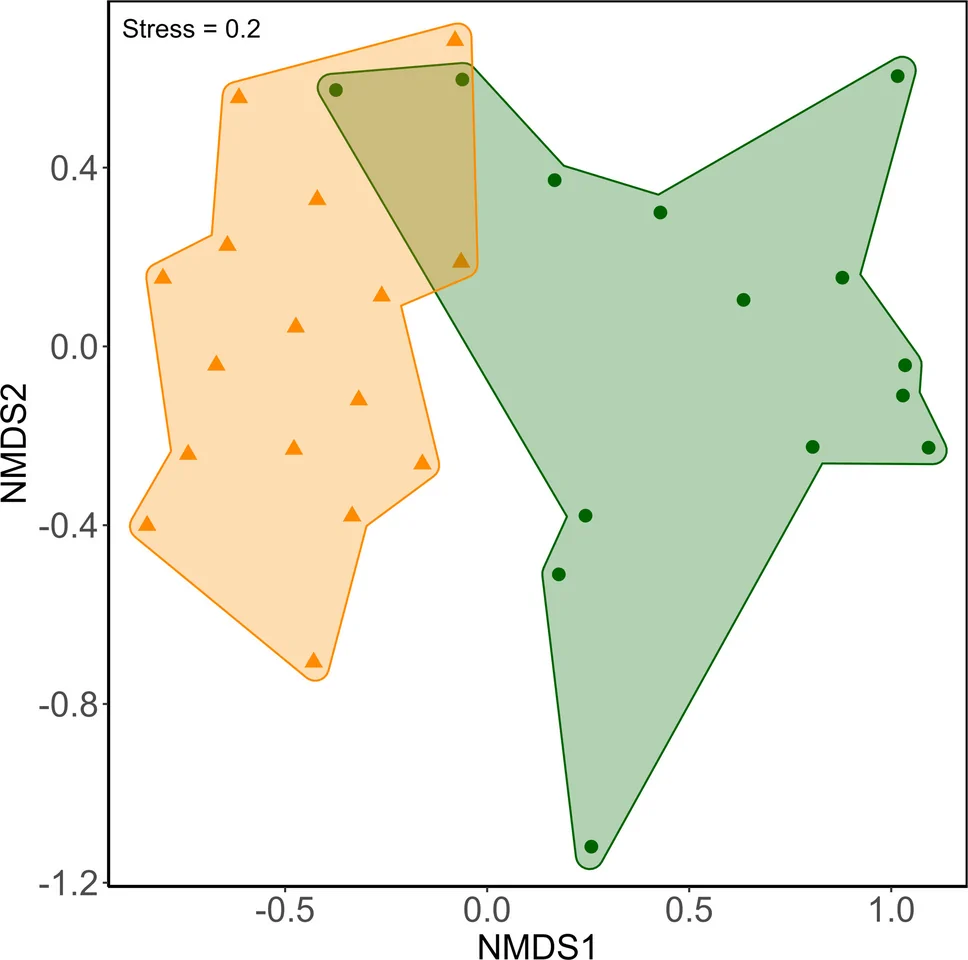
Non-metric multidimensional scaling (NMDS) plot based on Bray–Curtis dissimilarity (stress = 0.2, *k* = 2), with the species composition of carabid beetles in different habitats in mark hulls (orange and triangles urban, *n* = 16; green and circles rural, *n* = 14). Species abundances associated with both environments per site are available in Online Resource Table S6A

In total, 5,249 individual carabid beetles of 85 species were found across urban (*n* = 16) and rural (*n* = 14, 2 of the 16 sites did not achieve a sample coverage > 0.75) sites over four cities (Online Resource Table S4, S6A). Abundance was not significantly different in urban environments per site (183.44 ± 168.10) compared to rural (165.29 ± 111.62) (Generalised linear mixed model: β = 0.10 ± 0.25 SE, *z* = 0.413, *p* = 0.680), but species richness was lower (urban: 13.7 ± 4.51; rural: 19.4 ± 4.96) (Generalised linear mixed model: β = −0.35 ± 0.10 SE, *z* = −3.33, *p* = 0.0009). However, sample coverage differed among sites (Online Resource Table S4), so species richness comparisons should be interpreted with caution. The urban species composition differed significantly from the rural one (Adonis test: df = 1, *F* = 6.06, R^2^ = 0.18, *p* = 0.001) (Fig. 2). The ordination plot shows a stress level of 0.2, indicating an acceptable fit (Dexter et al. 2018).

### Trait composition

**Fig. 3:**
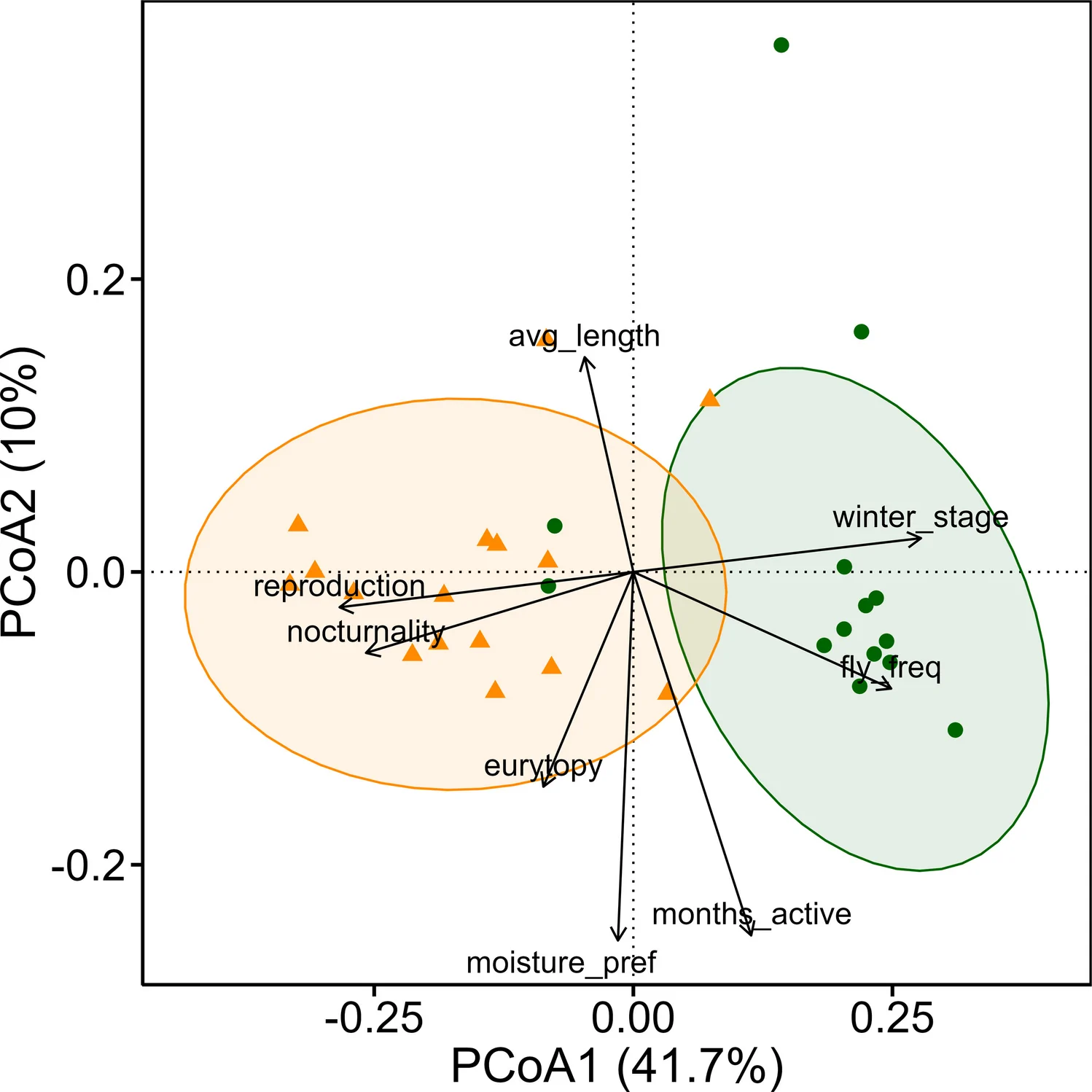
Principal coordinate analysis (PCoA) with Gower’s distance and Lingoes correction with the first two axes on the community weighted mean of eight traits. Traits were used for 85 carabid beetle species and sampled across the four replicated cities: avg_length (average body length), moisture_pref (habitat moisture preference), eurytopy (level of eurytopy), fly_freq (fly frequency), reproduction (timing of reproduction), winter_stage (overwintering stage), months_active (number of months active per year), and nocturnality (day or night active), respectively. Ellipses depict 95% confidence intervals, with colour indicating the habitat (orange: urban, green: rural). Urban sites (*n* = 16) are represented by orange triangles and rural sites (*n* = 14) by green circles

A PCoA on CWM of all carabid beetle traits showed that the first two axes capture a moderate amount of trait variation (Fig. 3). The trait composition differed significantly between both environments (Adonis test: df = 1, *F* = 29.10, R^2^ = 0.51, *p* = 0.001). Additionally, when comparing CWMs across cities within the same environment, trait values rarely differed significantly among the four cities we sampled, especially in the urban habitat (Online Resource Fig S3, Table S5). The first two PCoA axes had 51.7% of the cumulative proportion of variation (PCoA1: 41.7%; PCoA2: 10%). Urban communities were associated with autumn reproduction, more nocturnal activity, a higher level of eurytopy, more overwintering larvae than adults, but a lower fly frequency. In contrast, rural communities displayed opposite trait patterns. The five most important traits in explaining the whole ordination were timing of reproduction (r^2^ = 0.976), overwintering stage (r^2^ = 0.954), number of months active in the year (r^2^ = 0.900), nocturnality (r^2^ = 0.840) and fly frequency (r^2^ = 0.823).

### Functional diversity

**Fig. 4:**
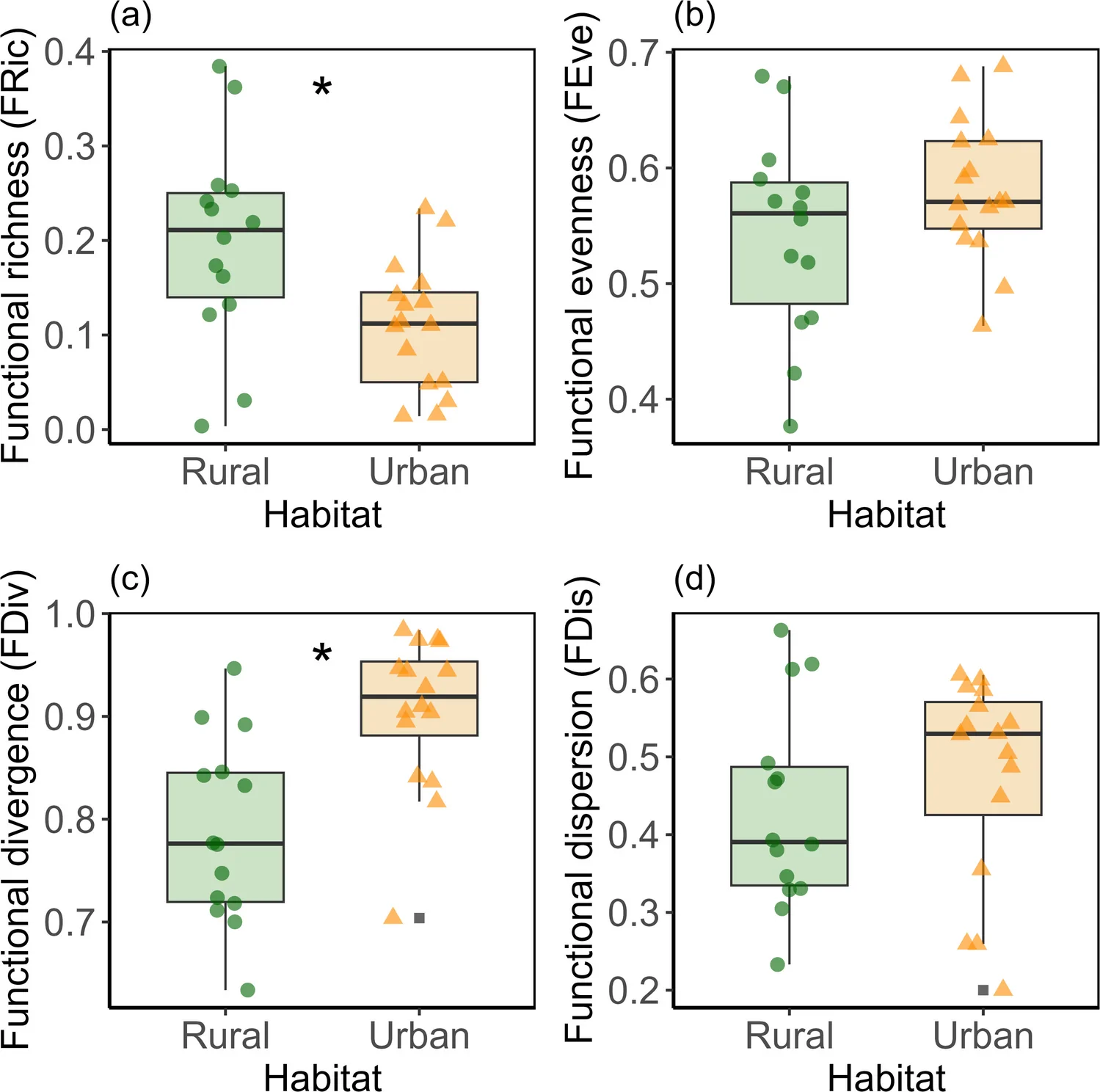
Functional diversity indices for carabid beetles in urban (orange, *n* = 16) and rural (green, *n* = 14) habitats. Box plots indicate the median and interquartile range. Functional diversity indices for a site are represented by orange triangles (urban) and green circles (rural). Black-filled squares indicate outliers. Asterisks indicate a significant difference in a functional diversity index between urban and rural habitats, with city taken into account as a random effect

Functional richness (FRic) was significantly lower in urban environments than in rural environments (Linear mixed model: β = −0.09 ± 0.03 SE, *z* = −2.74, *p* = 0.011), whereas functional divergence (FDiv) was significantly higher in urban environments than in rural ones (Generalised linear mixed model: β = 0.93 ± 0.22 SE, *z* = 4.16, *p* < 0.001) (Fig. 4a,c). However, functional evenness (FEve) and functional dispersion (FDis) were not significantly different between urban and rural environments (Generalised linear mixed model:β = 0.15 ± 0.09 SE, *z* = 1.72, *p* = 0.086; and Generalised linear mixed model: β = 0.04 ± 0.04 SE, *z* = 0.96, *p* = 0.338, respectively) (Fig. 4b,d).

## Discussion

Urban environments pose distinct challenges for invertebrates, acting as an environmental filter that shapes the composition of communities in cities. We demonstrate that urban carabid beetle compositions differed from their rural reference locations in both species composition and trait composition, with a higher species richness in the rural environment. Other studies investigating the relationship between species richness and habitat fragmentation found a similar result. In particular, increased impervious surface reduces connectivity and may limit species with lower dispersal capacity, thereby contributing to lower species richness in urban environments (Martinson and Raupp 2013; Beninde et al. 2015; Fenoglio et al. 2020). Moreover, urban carabid beetle communities converged on a distinct urban trait signature associated with a later timing of reproduction, higher proportion of larval overwintering, increased nocturnal activity, and lower fly frequency, while opposite trait patterns were found for rural communities. This suggests that the observed patterns in community composition in cities were primarily driven by environmental filtering, similar to previous research on urban carabid beetle assembly (Fournier et al. 2020), although a combination of environmental filtering and stochastic processes is also proposed as equally important (Magura et al. 2018; Perry et al. 2020). When evaluating functional structure at the community level, and treating urbanisation as the “disturbance” sensu Mouillot (2013), we observed the predicted reduction in urban functional richness (FRic), no effect on functional evenness or dispersion, and a significant increase in urban functional divergence (FDiv). This indicates that urbanisation shaped community trait composition towards narrower trait ranges and, simultaneously, to a higher frequency of trait values at the extremes of these ranges. For example, in our study, urban communities have extreme trait values, as shown by the strong tendency to reproduce late and be nocturnal. In line with previous studies, trait compositions differ (Piano et al. 2017; Hahs et al. 2023; Kotze et al. 2024), but this comparison seems to depend on the type of habitat used as the rural reference site. We discuss these findings and align them with the results from other cities and the theory of environmental filtering more generally, placing special emphasis on the unique habitats investigated in urban and rural environments. Specifically, we here emphasise the need to interpret findings as a function of both urbanisation and the selected habitat of the reference location (e.g., cities can be embedded, for instance, in a forest, marsh or pasture/grassland matrix and this may affect trait compositions). Moreover, we conceptually expand from interpreting urban sites simply as “disturbed” rural sites and consider they can comprise habitats very different from, and potentially more diverse, than rural habitats.

### Urban trait signatures

We show consistent trait variation across all four cities. More specifically, urban communities reproduced later in the year (i.e., early-mid August; aligning with the category “autumn breeders”), whereas rural communities reproduced earlier in the year (i.e., April; “spring breeders”). Autumn breeders generally overwinter as larvae, pupate in spring, and emerge as adults in summer, while spring breeders have larvae in summer, pupate in autumn, and overwinter as adults (Turin et al. 2022). Some late autumn breeding species (e.g., *Nebria brevicollis,* the most common urban species detected here) have a summer diapause, which could prevent damage caused by high summer temperatures, which are more pronounced in cities. Autumn breeders are also more frequently nocturnal (Tuf et al. 2012; Turin et al. 2022), which could be favourable in urban environments when associated with lower night temperatures, amount of cover and a higher predation risk by visual hunters (e.g., many birds).

Some of the results are in contrast to findings from studies that investigate urban trait signatures of carabid beetles in other cities. For example, Hahs et al. (2023) report predominantly adult overwintering in urban communities. Several other studies find higher dispersal ability in urban areas than in rural communities (Niemelä and Kotze 2009; Piano et al. 2017; Hahs et al. 2023; Kotze et al. 2024). Moreover, we found no strong difference in body size, level of eurytopy, or moisture preference. This contrasts with findings from other studies reporting that urban assemblages are typically composed of generalists and smaller specimens (Jones and Leather 2012), and strongly determined by moisture preference (Thiele 1977; van Dijk and den Boer 1992). This implies that urban trait signatures frequently vary across cities, which may be driven by the diversity of habitats or environmental conditions in urban areas.

Dispersal traits have repeatedly been associated with carabid beetle composition in urban environments (Niemelä and Kotze 2009; Perry et al. 2020), which is confirmed in our study. We and another study find evidence for lower dispersal abilities in urban beetle communities (based on wing morphology: Walker 2024, based on fly frequency: this study), whereas others with a higher dispersal ability in urban compared to rural communities (Niemelä and Kotze 2009; or a combination of both: Piano et al. 2017; based on wing morphology, not fly frequency: Hahs et al. 2023; Kotze et al. 2024). One explanation might be the choice of dispersal trait, for example fly frequency vs wing morphology (Fig. 3, Online Resource Fig S4). While fly frequency is based on observations of species truly flying, this does not necessarily hold for wing morphology. Obviously, brachypterous species cannot fly, but many macropterous species lack functional flight muscles and cannot fly either (Venn 2016). Desender (2000) mentions that habitat choice, evolution of life cycle timing and other life history traits explain the presence or absence of functional flight muscles, which may indicate that dispersal patterns based on wing morphology alone should be judged with caution. A second possible explanation for these mixed results may be the selected habitat of rural sites used to contrast with the urban environments. For example, knowing that dispersal trait compositions typically differ strongly between open grassland and forest habitats (Birkhofer et al. 2017; Zimmermann et al. 2024), urban trait signatures could fundamentally vary when non-urban reference locations are primarily situated in open grasslands and grassy road verges versus forests. For example, studies reporting urban beetles to have a higher dispersal ability than rural beetles compare cities to a rural forest matrix (Niemelä and Kotze 2009; Hahs et al. 2023; Kotze et al. 2024), while we compare to pastures. A third explanation relates to the expectation for the level of dispersal ability to match the accessibility and availability of suitable patches (Chowdhury et al. 2025). In highly fragmented urban areas, where reaching isolated patches has a low probability, selection may act against high dispersal (Cheptou et al. 2008; Finand et al. 2024; Walker 2024). In light of this, we encourage to use fly frequency over wing morphology as dispersal trait, report sampling time relative to the life cycle of species, and specify habitat characteristics of reference locations in more detail in future studies.

### Urban habitat characteristics

Our results of lower FRic and higher FDiv imply that fewer ecological strategies are required in urban environments, but within this narrower trait space, urban communities more commonly exhibit extreme trait values. This supports the prediction of Mouillot et al. (2013), that FRic decreases with increasing disturbance, but contradicts their prediction that FDiv would decline more rapidly. We agree with the suggestion of Mouillot et al. (2013) that FDiv may be better suited to detect community response to environmental change, as this metric is abundance-driven and likely to change before species are lost from communities. Yet, we argue that urban habitats frequently bear little resemblance to “disturbed” rural locations (e.g., through mowing, grazing, trampling, fertiliser and pesticide use, or drainage), but can comprise distinct, and potentially more diverse, habitats. We therefore suggest that FDiv is likely to increase frequently in response to urban environments, while this may not be true for simple “disturbances” in otherwise similar habitats.

Moreover, the different habitat types within urban and rural environments also play a role in determining trait composition. Although the difference in impervious surface is arguably an important and consistent distinction between our rural and urban sites, other differences may also have affected species and trait composition. For example, the lack of woody vegetation in some rural sites probably reduced the amount of dead wood and percentage canopy cover, which are significant determinants of carabid abundance and diversity (Fuller et al. 2008). Another factor we did not consider in our study is differences in vegetation composition between urban and rural environments. Although most carabid beetles do not rely on plants for food, the vegetation composition can still be important in shaping the trait composition through creation of microclimates.

### Conclusion

In this study, we showed that environmental filtering leads to consistent urban trait signatures in the four cities investigated here. Timing of reproduction, overwintering stage, nocturnality and fly frequency were most important in explaining the variation in carabid beetle traits composition between urban and rural environments. Species in cities predominantly bred in autumn, overwintered as larvae, were nocturnal and had a low fly frequency. At the same time, we highlight that urban trait signatures detected in other cities than our study, and/or using other habitats as rural reference locations, may vary in their respective trait composition. We emphasise the need to further investigate this context dependency based on habitat-specific effects on urban trait signatures, and their potential role in driving unexpected increases in functional divergence.

## Supplementary Information

Below is the link to the electronic supplementary material.

Supplementary file1 (PDF 1380 KB)

## Data Availability

The data and scripts that support the findings of this study are openly available in YODA at https://doi.org/10.48338/VU01-XG27JV.
