# Supplementary material for: Carabid beetle traits tell tales of urban filtering: shifts in activity, flight behaviour, reproduction, and overwintering
Source: Urban Ecosyst. 2026 Sep 15;29(5):268. doi: 10.1007/s11252-026-02128-8 (PMC13577967; doi:10.1007/s11252-026-02128-8)
Supplement: Supplementary file 1 [file 11252_2026_2128_MOESM1_ESM.pdf]

**Carabid beetle traits tell tales of urban filtering: shifts in activity, flight behaviour, reproduction, and overwintering**

*Online resource material*

Urban Ecosystems

Wouter B. van der Vegt[1], Jacintha Ellers[1], Nienke A. Schijfsma[1], Olaf T. van der Woude[1], Joscha Beninde[1], Matty P. Berg[1,2]

[1]Section Ecology & Evolution, A-LIFE, Vrije Universiteit Amsterdam, Amsterdam, The Netherlands

[2]Conservation Ecology Group, GELIFES, University of Groningen, Groningen, The Netherlands

Corresponding author: Wouter B. van der Vegt

Address: De Boelelaan 1108, 1081 HZ Amsterdam, The Netherlands

Email: w.b.van.der.vegt@vu.nl

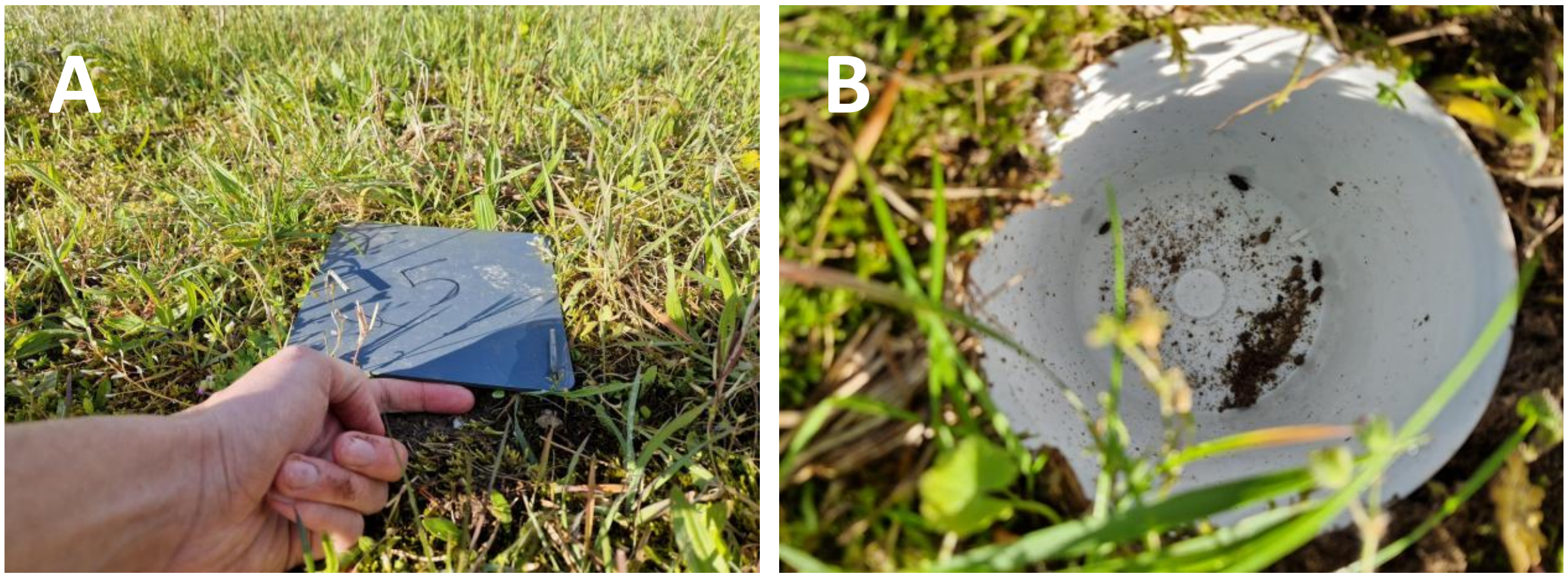


**Fig S1** Setup of pitfall traps. Pitfall traps (polypropylene cups, depth = 12 cm, diameter = 11 cm) were placed in the soil with the opening of the trap at soil surface level. Each trap was covered from sunlight, rainfall and debris by grey polyvinyl chloride (PVC) roofs (17 x 17 cm). Roofs were placed approximately two centimetres above the trap opening and were secured with galvanised pegs (diameter = 3.6 mm). Rubber O-rings (outer diameter = 7.8 mm, inner diameter = 3.2 mm, thickness = 2.3 mm) were placed around the pegs underneath the roof, so the roof remained at the same level. Panel A shows the setup as used in the field with the margin of space between the soil surface and the roof, which is approximately two cm or one human finger. Panel B shows the setup before adding the saturated saline solution of cooking salt and tap water and placing the roof accordingly just above the trap

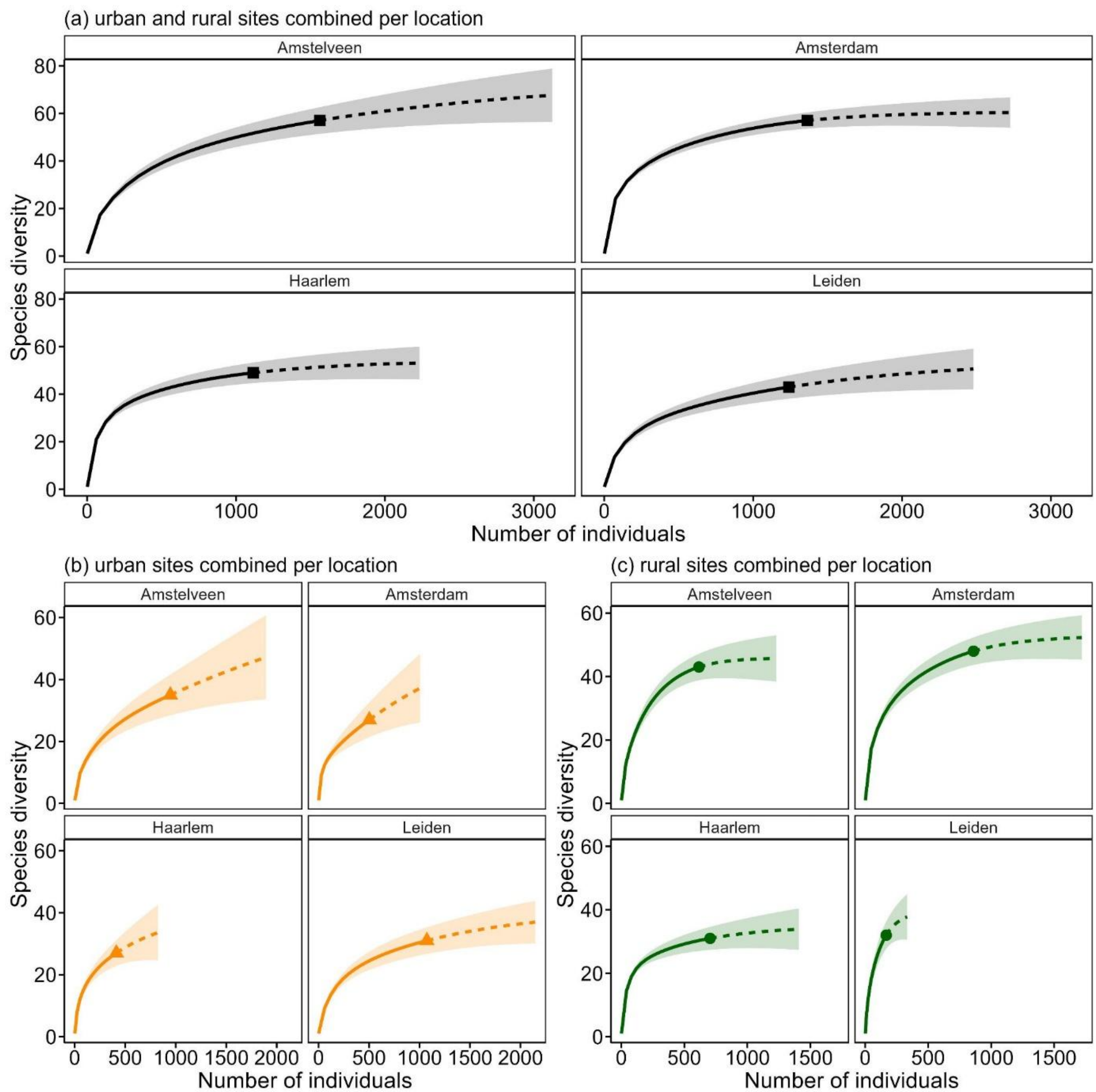


**Fig S2** Sample-size-based rarefaction (solid line) and extrapolation (dotted line) curves with 95% confidence intervals (shaded areas) for carabid beetle species richness for four locations (Amstelveen, Amsterdam, Haarlem and Leiden) in two habitats (urban and rural). (a) in black shows the curves when urban and rural sites are taken together per city, (b) in orange only for urban sites per location represented, and (c) in green only for rural sites per location

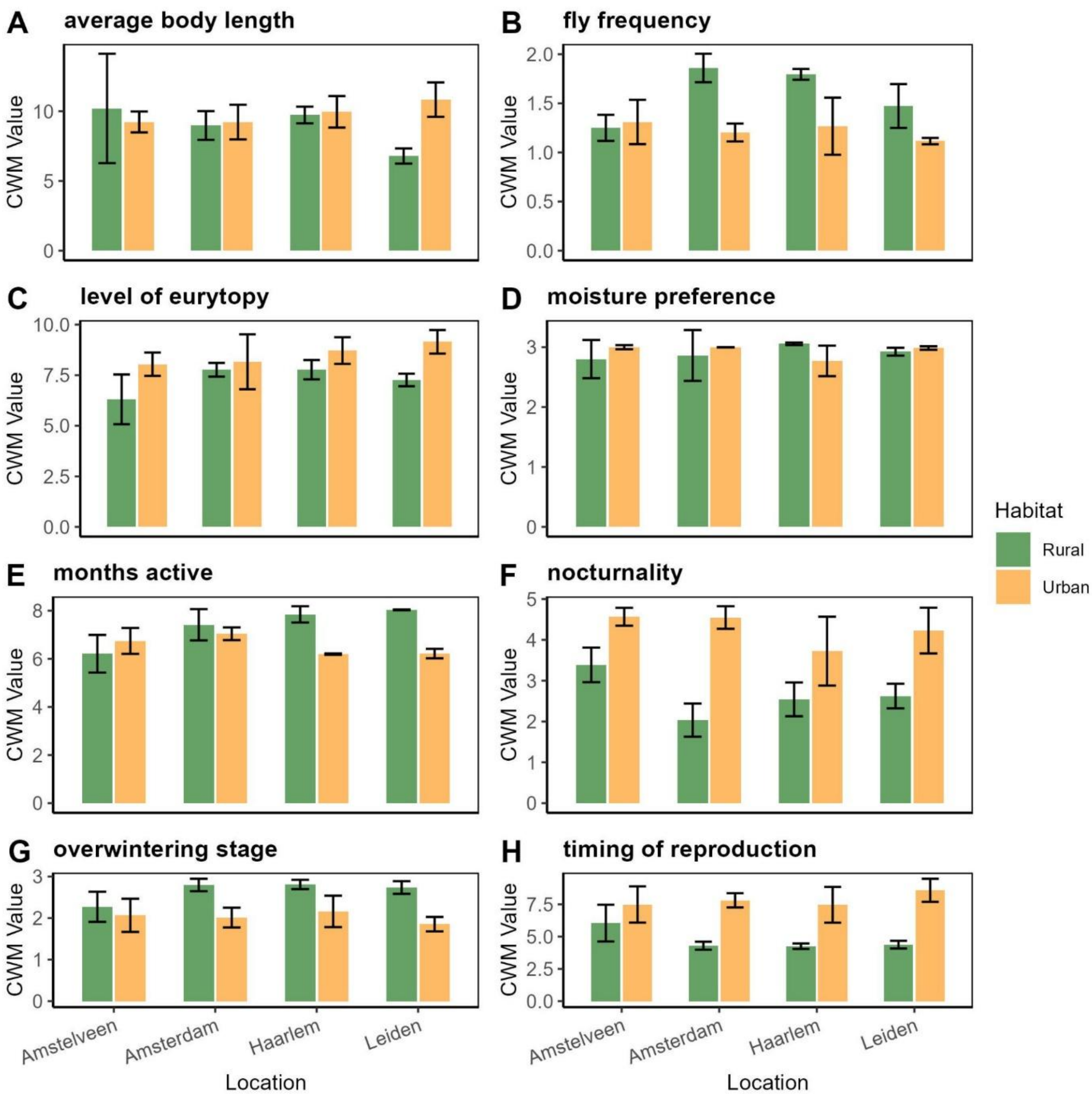


**Fig S3** Barplots with the community weighted mean (CWM) trait values for eight traits across locations. (A) Average body length, with higher CWM values indicating a larger average body size. (B) Fly frequency, with CWM values around 1 indicating communities that never or rarely fly and values around 2 frequently fly. (C) Level of eurytopy, with higher CWM values indicating a more generalist community. (D) Moisture preference, where higher CWM values indicate a preference for more moist conditions, lower CWM values for drier conditions, and values around 3 for no preference. (E) Months active, where higher CWM values indicate that

communities are longer active in the year. (F) Nocturnality, where CWM values around 5 indicate nocturnal communities, values around 3 indicate day-night communities and values around 1 indicate diurnal communities. (G) Overwintering stage, where CWM values around 3 indicate that individuals from this community mostly overwinter as adults, values around 2 as larvae and adults, and values around 1 as larvae. (H) Timing of reproduction, where higher CWM values indicate later reproduction in the year. See also Table 1 for a complete explanation of the traits. Green bars indicate the CWM for rural sites per location (n = 4 per location, but n = 3 for Amstelveen and Leiden due to low sample coverage) and orange bars the urban sites (n = 4 per location). Error bars represent the standard deviation. See Table S5 for statistical output

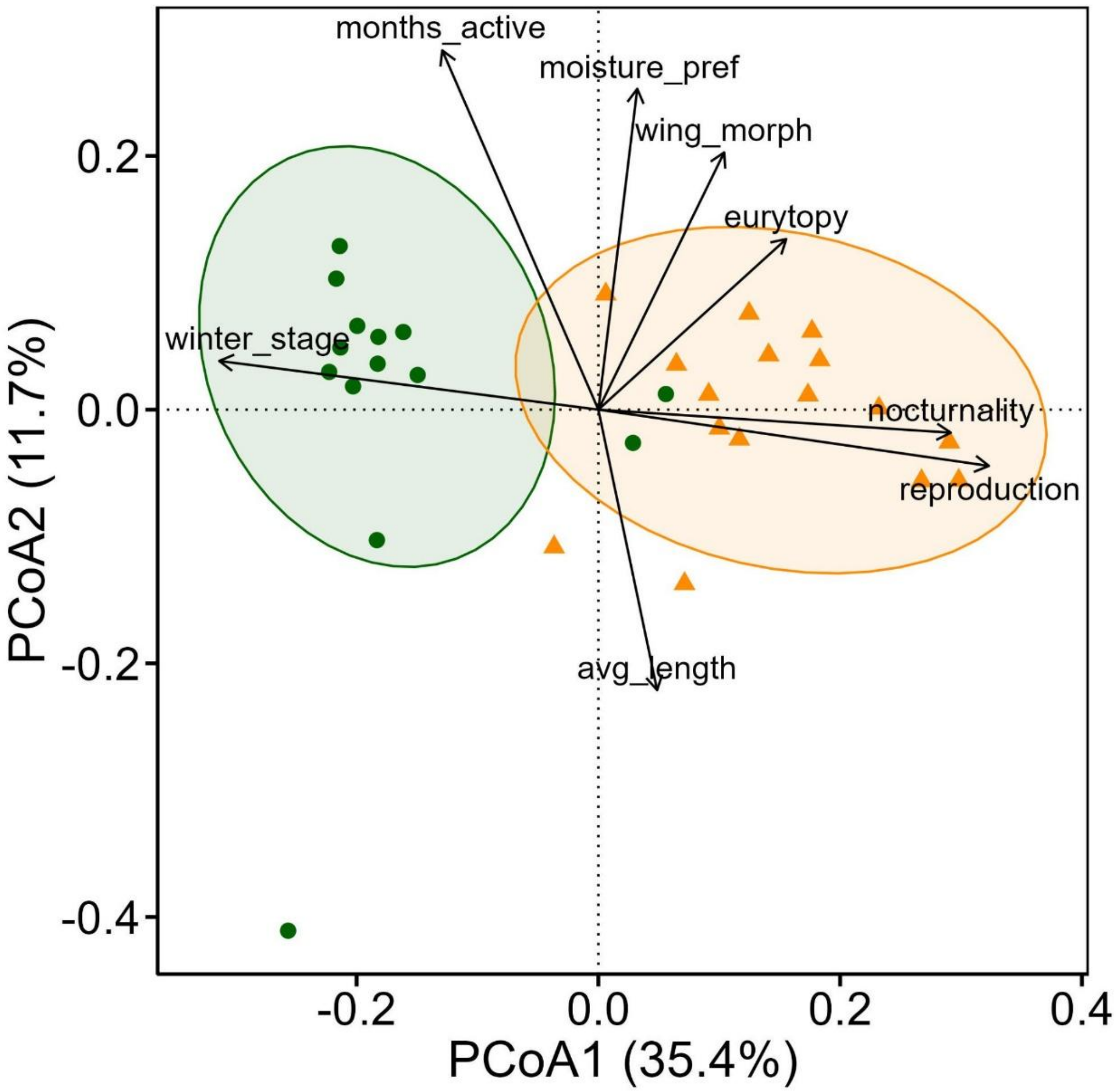


**Fig S4** Principal Coordinate Analysis (PCoA) with Gower's distance and Lingoes correction with the first two axes (combined they explained 47.1% of the variation) on the community weighted mean of eight traits. Traits were used for 85 carabid beetle species and sampled across the four replicated cities: avg_length (average body length), moisture_pref (habitat moisture preference), eurytopy (level of eurytopy), wing_morph (wing morphology; as described by Dutch literature and identification keys (Turin 2000, Boeken et al. 2002, Muilwijk et al. 2015), reproduction (timing of reproduction), winter_stage (overwintering stage), months_active (number of months active per year), and nocturnality (day or night active), respectively. Ellipses

depict a 95% confidence interval, with the colour determining the habitat (orange: urban, green: rural). Urban sites (n = 16) are represented by orange triangles and rural sites (n = 14) by green circles.  The trait composition differed significantly between both environments (Adonis test: df = 1, F = 26.87, $R^2$ = 0.49, p = 0.001)

**Table S1** Descriptive summary of the cities and sites visited. Location represents the chosen cities and site the selected trapping places within or adjacent to the city. Habitat displays whether a site was urban or rural (see Materials and Methods for description). Latitude and longitude resemble the coordinates in decimal degrees of the sites. At each site five pitfall traps were placed at minimum 5 m distance (preferably 10 m) and were fortnightly emptied and refilled with new fixation fluid (saturated salt water solution of tap water with cooking salt). Five rounds of pitfall trapping was performed (i.e., in total 10 weeks of samples were collected)

| **Location** | **Site** | **Habitat** | **Latitude** | **Longitude** |
|---|---|---|---|---|
| Amstelveen | Lelyplantsoen | Urban | 52.30507 | 4.85742 |
| Amstelveen | VanPrinsterenlaan | Urban | 52.3052 | 4.86354 |
| Amstelveen | Meanderpark | Urban | 52.30134 | 4.85992 |
| Amstelveen | Stadstuinen | Urban | 52.30201 | 4.86721 |
| Amstelveen | Polderweg_west | Rural | 52.29007 | 4.90396 |
| Amstelveen | Polderweg east | Rural | 52.28817 | 4.91182 |
| Amstelveen | Rondehoep_east | Rural | 52.28089 | 4.92076 |
| Amstelveen | In_de_schuur | Rural | 52.27544 | 4.92457 |
| Amsterdam | Weteringschans | Urban | 52.359668 | 4.888727 |
| Amsterdam | Frederiksplein | Urban | 52.360436 | 4.899077 |

| Location | Site | Habitat | Latitude | Longitude |
|---|---|---|---|---|
| Amsterdam | Wertheimpark | Urban | 52.367989 | 4.909145 |
| Amsterdam | Marineterrein | Urban | 52.372431 | 4.916997 |
| Amsterdam | Golfbaan | Rural | 52.401892 | 4.958044 |
| Amsterdam | Zunderdorp_south | Rural | 52.412043 | 4.969912 |
| Amsterdam | Zunderdorp_north | Rural | 52.417184 | 4.980389 |
| Amsterdam | Broek_in_Waterland | Rural | 52.423899 | 4.991668 |
| Haarlem | Wijngaardtuin | Urban | 52.38224392 | 4.637359739 |
| Haarlem | Egelantierstuin | Urban | 52.377342 | 4.634734 |
| Haarlem | Proveniershof | Urban | 52.377342 | 4.630846 |
| Haarlem | Kenaupark | Urban | 52.387597 | 4.632345 |
| Haarlem | OEV5 | Rural | 52.39037 | 4.69844 |
| Haarlem | OEV7 | Rural | 52.40014 | 4.70007 |
| Haarlem | OEV13 | Rural | 52.40325 | 4.68227 |
| Haarlem | OEV16 | Rural | 52.39407 | 4.7134 |
| Leiden | Rembrandtpark | Urban | 52.160152 | 4.481228 |
| Leiden | Plantsoen | Urban | 52.153601 | 4.495562 |

| Location | Site | Habitat | Latitude | Longitude |
|---|---|---|---|---|
| Leiden | VanDerLubbehof | Urban | 52.157495 | 4.497341 |
| Leiden | Blekerspark | Urban | 52.162921 | 4.501910 |
| Leiden | h4.1 | Rural | 52.178893 | 4.559852 |
| Leiden | h5.0 | Rural | 52.183791 | 4.570610 |
| Leiden | h5.5 | Rural | 52.184834 | 4.577402 |
| Leiden | h6.1 | Rural | 52.186074 | 4.586006 |

**Table S2** The average annual temperature (in °C) and rainfall (in mm) per city (source: https://en.climate-data.org/). Standard deviation was not available. Data was retrieved in February 2026

| Location | Average annual temperature (°C) | Average annual rainfall (mm) |
|---|---|---|
| Amstelveen | 10.6 | 930 |
| Amsterdam | 10.7 | 844 |
| Haarlem | 10.8 | 915 |
| Leiden | 10.8 | 940 |

**Table S3** Summary table with all trait values per species used in this study. Column names and reference origins are explained in Table 1. Asterisks indicate imputed trait values that were based on known trait values, with more weight on the genus to impute the unknown/missing values. Imputation was performed using Multivariate Imputation by Chained Equations (MICE) v3.18.0 with predictive mean matching and 10 imputations (Buuren and Groothuis-Oudshoorn 2011)

| **Species** | **avg_length** | **eurytopy** | **fly_freq** | **moisture_pref** | **months_active** | **nocturnality** | **reproduction** | **winter_stage** |
|---|---|---|---|---|---|---|---|---|
| *Acupalpus dubius* | 2.7 | 3 | 2 | 5 | 6 | 1 | 4 | 3 |
| *Acupalpus exiguus* | 2.75 | 2 | 2 | 4 | 6 | 3 | 4 | 3 |
| *Acupalpus parvulus* | 3.6 | 6 | 2 | 5 | 4 | 1 | 4 | 3 |
| *Agonum muelleri* | 8.2 | 9 | 2 | 3 | 9 | 1 | 4 | 3 |
| *Agonum viduum* | 8.3 | 5 | 1 | 3 | 7 | 1 | 4 | 3 |
| *Amara aenea* | 7.5 | 9 | 2 | 3 | 7 | 1 | 4 | 3 |

| **Species** | **avg_length** | **eurytopy** | **fly_freq** | **moisture_pref** | **months_active** | **nocturnality** | **reproduction** | **winter_stage** |
|---|---|---|---|---|---|---|---|---|
| *Amara anthobia* | 6.25 | 5 | 2 | 2 | 6 | 1 | 4 | 3.0 * |
| *Amara bifrons* | 6.4 | 8 | 2 | 3 | 5 | 5 | 10 | 1 |
| *Amara communis* | 8 | 10 | 2 | 3 | 8 | 1 | 4 | 2 |
| *Amara convexior* | 8 | 8 | 1 | 2 | 6 | 1 | 4 | 3 |
| *Amara curta* | 6.65 | 6 | 1 | 2 | 4 | 1 | 4 | 3 |
| *Amara familiaris* | 6.5 | 9 | 2 | 3 | 8 | 1 | 4 | 3 |
| *Amara fulva* | 9.25 | 7 | 1 | 3 | 5 | 4 | 10 | 2 |
| *Amara lucida* | 5.7 | 5 | 2 | 2 | 4 | 1 | 5.5 | 3 |
| *Amara lunicollis* | 7.75 | 8 | 2 | 3 | 8 | 1 | 4 | 3 |
| *Amara ovata* | 8.75 | 6 | 1 | 3 | 5 | 1 | 4 | 3 |
| *Amara plebeja* | 7 | 9 | 2 | 3 | 8 | 1 | 4 | 3 |

| Species | avg_length | eurytopy | fly_freq | moisture_pref | months_active | nocturnality | reproduction | winter_stage |
|---|---|---|---|---|---|---|---|---|
| *Amara similata* | 8.75 | 7 | 2 | 3 | 7 | 1 | 4 | 2 |
| *Amara spreta* | 8.25 | 9 | 1 | 3 | 6 | 1 | 4 | 3 |
| *Anchomenus dorsalis* | 7 | 7 | 2 | 3 | 8 | 5 | 4 | 3 |
| *Anisodactylus binotatus* | 11 | 7 | 2 | 3 | 7 | 4 | 4 | 3 |
| *Asaphidion curtum* | 4.15 | 0 | 2 | 3 | 7 | 1 | 4 | 2.0 * |
| *Asaphidion flavipes* | 4.4 | 8 | 2 | 3 | 7 | 1 | 4 | 2 |
| *Badister bullatus* | 6.15 | 8 | 1 | 3 | 9 | 4 | 5.5 | 3 |

| Species | avg_length | eurytopy | fly_freq | moisture_pref | months_active | nocturnality | reproduction | winter_stage |
|---|---|---|---|---|---|---|---|---|
| *Badister lacertosus* | 6 | 6 | 1 | 3 | 9 | 4 | 4 | 3 |
| *Badister sodalis* | 4.25 | 5 | 1 | 3 | 7 | 4 | 4 | 3 |
| *Badister unipustulatus* | 8 | 5 | 2 | 3 | 9 | 3 | 5.5 | 3 |
| *Bembidion biguttatum* | 4 | 5 | 2 | 3 | 9 | 1 | 4 | 3 |
| *Bembidion guttula* | 3.15 | 8 | 1 | 3 | 11 | 1 | 4 | 3 |
| *Bembidion lampros* | 3.5 | 9 | 2 | 3 | 8 | 2 | 4 | 3 |

| Species | avg_length | eurytopy | fly_freq | moisture_pref | months_active | nocturnality | reproduction | winter_stage |
|---|---|---|---|---|---|---|---|---|
| *Bembidion lunulatum* | 3.55 | 6 | 2 | 4 | 11 | 1 | 4 | 3 |
| *Bembidion obtusum* | 3.2 | 5 | 1 | 3 | 11 | 3 | 4 | 3 |
| *Bembidion properans* | 4 | 8 | 2 | 3 | 8 | 1 | 5.5 | 3 |
| *Bembidion quadrimaculatum* | 3.15 | 7 | 2 | 3 | 11 | 3 | 4 | 3 |
| *Bembidion tetracolum* | 5.5 | 8 | 1 | 3 | 9 | 1 | 4 | 3 |
| *Bradycellus harpalinus* | 4.4 | 9 | 2 | 3 | 11 | 5 | 9 | 1 |

| Species | avg_length | eurytopy | fly_freq | moisture_pref | months_active | nocturnality | reproduction | winter_stage |
|---|---|---|---|---|---|---|---|---|
| *Bradycellus verbasci* | 5 | 7 | 2 | 3 | 6 | 5 | 9 | 2 |
| *Calathus cinctus* | 7.5 | 4 | 2 | 2 | 7 | 5 | 10 | 2 |
| *Calathus melanocephalus* | 7.5 | 10 | 1 | 3 | 8 | 5 | 8.5 | 2 |
| *Calathus rotundicollis* | 9.75 | 8 | 1 | 3 | 10 | 5 | 8.5 | 1 |
| *Carabus auratus* | 23.5 | 3 | 1 | 2 | 3 | 1 | 4 | 3 |
| *Carabus granulatus* | 19.5 | 7 | 1 | 3 | 7 | 4 | 4 | 3 |
| *Carabus nemoralis* | 23 | 8 | 1 | 3 | 8 | 4 | 4 | 3 |

| Species | avg_length | eurytopy | fly_freq | moisture_pref | months_active | nocturnality | reproduction | winter_stage |
|---|---|---|---|---|---|---|---|---|
| *Clivina collaris* | 5 | 6 | 1 | 3 | 4 | 5 | 5.5 | 3 |
| *Clivina fossor* | 5.75 | 9 | 2 | 3 | 6 | 5 | 4 | 3 |
| *Demetrias atricapillus* | 5.25 | 4 | 2 | 3 | 9 | 3 | 4 | 3 |
| *Dyschirius globosus* | 2.5 | 9 | 1 | 3 | 7 | 1 | 4 | 3 |
| *Harpalus affinis* | 10.5 | 9 | 2 | 3 | 6 | 4 | 5 | 2.5 |
| *Harpalus anxius* | 7.5 | 5 | 1 | 1 | 6 | 5 | 5 | 2.5 |
| *Harpalus latus* | 9.5 | 8 | 1 | 3 | 5 | 1 | 5 | 2 |
| *Harpalus luteicornis* | 6.75 | 3 | 1 | 2 | 5 | 1 | 4 | 3 |

| Species | avg_length | eurytopy | fly_freq | moisture_pref | months_active | nocturnality | reproduction | winter_stage |
|---|---|---|---|---|---|---|---|---|
| *Harpalus rubripes* | 9.6 | 5 | 1 | 2 | 5 | 5 | 9 | 1.5 |
| *Harpalus rufipes* | 13.35 | 10 | 2 | 3 | 5 | 5 | 10 | 2 |
| *Harpalus tardus* | 9.4 | 8 | 2 | 2 | 6 | 1 | 5 | 3 |
| *Leistus ferrugineus* | 6.75 | 8 | 1 | 2 | 5 | 5 | 10 | 1 |
| *Leistus fulvibarbis* | 7 | 4 | 1 | 3 | 6 | 5 | 10 | 1.0 * |
| *Leistus rufomarginatus* | 8.5 | 7 | 1 | 3 | 5 | 5 | 10 | 1 |
| *Loricera pilicornis* | 7.25 | 10 | 2 | 3 | 7 | 4 | 4 | 2.5 |

| Species | avg_length | eurytopy | fly_freq | moisture_pref | months_active | nocturnality | reproduction | winter_stage |
|---|---|---|---|---|---|---|---|---|
| *Nebria brevicollis* | 12 | 10 | 1 | 3 | 6 | 5 | 10 | 1.5 |
| *Notiophilus biguttatus* | 4.75 | 9 | 1 | 3 | 7 | 1 | 4 | 3 |
| *Notiophilus palustris* | 5 | 8 | 1 | 3 | 7 | 1 | 4 | 3 |
| *Notiophilus rufipes* | 5.25 | 7 | 1 | 3 | 5 | 1 | 4 | 3 |
| *Notiophilus substriatus* | 4.75 | 7 | 1 | 3 | 5 | 1 | 4 | 3.0 * |
| *Oodes helopioides* | 8.75 | 5 | 1 | 3 | 8 | 2 | 4 | 3 |

| Species | avg_length | eurytopy | fly_freq | moisture_pref | months_active | nocturnality | reproduction | winter_stage |
|---|---|---|---|---|---|---|---|---|
| *Ophonus rufibarbis* | 8.25 | 5 | 2 | 2 | 5 | 5 | 4 | 1 |
| *Oxypselaphus obscurus* | 5.7 | 8 | 1 | 3 | 11 | 5 | 4 | 2 |
| *Panagaeus bipustulatus* | 7 | 6 | 1 | 2 | 4 | 1 | 4 | 3 |
| *Paranchus albipes* | 7.9 | 4 | 1 | 4 | 8 | 5 | 9 | 2 |
| *Poecilus cupreus* | 12.25 | 7 | 2 | 3 | 7 | 1 | 4 | 2.5 |
| *Poecilus versicolor* | 11.5 | 9 | 2 | 3 | 8 | 1 | 4 | 3 |

| Species | avg_length | eurytopy | fly_freq | moisture_pref | months_active | nocturnality | reproduction | winter_stage |
|---|---|---|---|---|---|---|---|---|
| *Pterostichus anthracinus* | 10.75 | 4 | 1 | 3 | 7 | 5 | 4 | 3 |
| *Pterostichus diligens* | 5.6 | 8 | 2 | 3 | 8 | 4 | 4 | 2.5 |
| *Pterostichus melanarius* | 15.5 | 9 | 1 | 3 | 7 | 5 | 10 | 2 |
| *Pterostichus minor* | 7.35 | 7 | 1 | 4 | 9 | 4 | 4 | 3 |
| *Pterostichus nigrita* | 10.25 | 9 | 2 | 3 | 7 | 4 | 4 | 3 |
| *Pterostichus oblongopunctatus* | 10.8 | 7 | 1 | 3 | 9 | 3 | 4 | 3 |

| Species | avg_length | eurytopy | fly_freq | moisture_pref | months_active | nocturnality | reproduction | winter_stage |
|---|---|---|---|---|---|---|---|---|
| *Pterostichus strenuus* | 6.1 | 9 | 2 | 3 | 10 | 4 | 4 | 3 |
| *Pterostichus vernalis* | 6.75 | 9 | 2 | 3 | 11 | 5 | 4 | 3 |
| *Stenolophus mixtus* | 5.5 | 6 | 2 | 3 | 7 | 5 | 4 | 3 |
| *Stenolophus teutonus* | 6.25 | 2 | 2 | 3 * | 5 | 5 | 4 | 3 |
| *Stomis pumicatus* | 7.65 | 6 | 1 | 3 | 9 | 5 | 5.5 | 3 |
| *Syntomus foveatus* | 3.45 | 8 | 2 | 3 | 5 | 2 | 4 | 3 |

| Species | avg_length | eurytopy | fly_freq | moisture_pref | months_active | nocturnality | reproduction | winter_stage |
|---|---|---|---|---|---|---|---|---|
| *Trechoblemus micros* | 4.5 | 6 | 1 | 3 | 11 | 5 | 5.5 | 3 |
| *Trechus obtusus* | 3.75 | 8 | 2 | 3 | 11 | 4 | 9 | 1 |
| *Trechus quadristriatus* | 4 | 8 | 2 | 3 | 6 | 5 | 9 | 1.5 |

**Table S4** Table with the iNEXT sample-sized based rarefaction/extrapolation analyses output (Hsieh et al. 2016). The column "Habitat and location" indicates for which habitat (either urban, rural or both) and location (Amstelveen, Amsterdam, Haarlem or Leiden) the rarefaction curves were made, "n" for the number of individuals caught, "Species observed" for the observed species richness and "Sample coverage" for the completeness of sampling in proportions. Asterisks signify sites that have been omitted from the analysis due to low sample coverage, but are shown here for completeness

| Habitat and location | n | Species observed | Sample coverage |
|---|---|---|---|
| *Amstelveen rural* | | | |
| In_de_Schuur | 8 | 6 | 0.5625* |
| Polderweg_east | 127 | 23 | 0.9375 |
| Polderweg_west | 71 | 23 | 0.8471 |
| Rondehoep_east | 409 | 20 | 0.9878 |
| *Amstelveen urban* | | | |
| Lelyplantsoen | 123 | 10 | 0.9840 |
| Meanderpark | 287 | 21 | 0.9792 |
| Van_Prinsterenlaan | 93 | 15 | 0.9250 |
| Stadstuinen | 444 | 18 | 0.9798 |
| *Amsterdam rural* | | | |
| Broek_in_Waterland | 308 | 23 | 0.9741 |
| Golfbaan | 184 | 18 | 0.9676 |

| Habitat and location | n | Species observed | Sample coverage |
|---|---|---|---|
| Zunderdorp_north | 109 | 11 | 0.9915 |
| Zunderdorp_south | 259 | 23 | 0.9847 |
| *Amsterdam urban* | | | |
| Frederiksplein | 94 | 10 | 0.9577 |
| Marineterrein | 165 | 16 | 0.9516 |
| Wertheimpark | 149 | 12 | 0.9868 |
| Weteringschans | 95 | 14 | 0.9583 |
| *Haarlem rural* | | | |
| OEV5 | 86 | 18 | 0.9548 |
| OEV7 | 265 | 23 | 0.9737 |
| OEV13 | 149 | 19 | 0.9666 |
| OEV16 | 204 | 26 | 0.9609 |
| *Haarlem urban* | | | |
| Egelantierstuin | 126 | 10 | 0.9525 |
| Kenaupark | 139 | 15 | 0.9500 |
| Proveniershof | 90 | 15 | 0.9563 |
| Wijngaardtuin | 57 | 10 | 0.9480 |
| *Leiden rural* | | | |
| h4.1 | 43 | 17 | 0.7707 |
| h5.0 | 46 | 8 | 0.9592 |

| Habitat and location | n | Species observed | Sample coverage |
|---|---|---|---|
| h5.5 | 24 | 11 | 0.5873* |
| h6.1 | 54 | 19 | 0.7600 |
| *Leiden urban* | | | |
| Blekerspark | 696 | 23 | 0.9914 |
| Plantsoen | 152 | 15 | 0.9476 |
| Rembrandtpark | 34 | 8 | 0.8547 |
| Van_der_Lubbehof | 191 | 7 | 0.9843 |

**Table S5** Statistical results for pairwise comparisons of community weighted means (CWMs) of eight traits among locations within each habitat (see Figure S3 for the associated traits). Since each site provided one CWM value (i.e. no replication on site level), linear models were fitted as lm(*trait ~ Habitat * Location)*. Then, pairwise comparisons among locations within habitats were done using estimated marginal means with Tukey-adjust post hoc tests via the emmeans package (emmeans*(model, pairwise ~ Location | Habitat)*). For moisture preference, model assumptions were violated, so differences among locations were tested separately within urban and rural habitats with Kruskal-Wallis tests followed by Dunn post hoc tests with Bonferroni adjustment. In the models, Trait represents CWM values per site, Habitat for either urban or rural samples, and Location the cities (Amstelveen, Amsterdam, Haarlem, Leiden). In the table, Trait represents the investigated trait. Comparison the pairwise comparison. Estimate the estimated difference between group means. SE the standard error of this estimate. df the degrees of freedom of the test. t / z the t-ratio (for emmeans results of linear models) or z-score (for Dunn tests; only for the trait "moisture preference") of the comparison. p whether a comparison was significantly different (i.e., $p < 0.05$). Significant differences are made bold

| **Trait** | **Comparison** | **Habitat** | **Estimate** | **SE** | **df** | **t / z** | **p** |
|---|---|---|---|---|---|---|---|
| Average body length | Amstelveen - Amsterdam | Rural | 1.224 | 1.155 | 22 | 1.06 | 0.718 |
| | Amstelveen - Haarlem | | 0.469 | 1.155 | 22 | 0.41 | 0.977 |

| Trait | Comparison | Habitat | Estimate | SE | df | t / z | p |
|---|---|---|---|---|---|---|---|
| | Amstelveen - Leiden | | 3.411 | 1.235 | 22 | 2.76 | 0.052 |
| | Amsterdam - Haarlem | | -0.754 | 1.070 | 22 | -0.70 | 0.894 |
| | Amsterdam - Leiden | | 2.188 | 1.155 | 22 | 1.89 | 0.259 |
| | Haarlem - Leiden | | 2.942 | 1.155 | 22 | 2.55 | 0.080 |
| | Amstelveen - Amsterdam | Urban | 0.009 | 1.070 | 22 | 0.01 | 1 |
| | Amstelveen - Haarlem | | -0.727 | 1.070 | 22 | -0.68 | 0.903 |
| | Amstelveen - Leiden | | -1.600 | 1.070 | 22 | -1.50 | 0.457 |
| | Amsterdam - Haarlem | | -0.736 | 1.070 | 22 | -0.69 | 0.900 |
| | Amsterdam - Leiden | | -1.609 | 1.070 | 22 | -1.50 | 0.452 |
| | Haarlem - Leiden | | -0.873 | 1.070 | 22 | -0.82 | 0.846 |
| Fly frequency | Amstelveen - Amsterdam | Rural | -0.609 | 0.130 | 22 | -4.67 | **0.001** |

| Trait | Comparison | Habitat | Estimate | SE | df | t / z | p |
|---|---|---|---|---|---|---|---|
| | Amstelveen - Haarlem | | -0.545 | 0.130 | 22 | -4.18 | **0.002** |
| | Amstelveen - Leiden | | -0.223 | 0.139 | 22 | -1.56 | 0.401 |
| | Amsterdam - Haarlem | | 0.065 | 0.121 | 22 | 0.54 | 0.949 |
| | Amsterdam - Leiden | | 0.387 | 0.130 | 22 | 2.97 | **0.033** |
| | Haarlem - Leiden | | 0.322 | 0.130 | 22 | 2.47 | 0.093 |
| | Amstelveen - Amsterdam | Urban | 0.107 | 0.121 | 22 | 0.89 | 0.810 |
| | Amstelveen - Haarlem | | 0.043 | 0.121 | 22 | 0.36 | 0.984 |
| | Amstelveen - Leiden | | 0.195 | 0.121 | 22 | 1.61 | 0.392 |
| | Amsterdam - Haarlem | | -0.064 | 0.121 | 22 | -0.53 | 0.951 |
| | Amsterdam - Leiden | | 0.087 | 0.121 | 22 | 0.72 | 0.887 |
| | Haarlem - Leiden | | 0.151 | 0.121 | 22 | 1.25 | 0.601 |

| Trait | Comparison | Habitat | Estimate | SE | df | t / z | p |
|---|---|---|---|---|---|---|---|
| Level of eurytopy | Amstelveen - Amsterdam | Rural | -1.46 | 0.589 | 22 | -2.47 | 0.090 |
| | Amstelveen - Haarlem | | -1.47 | 0.589 | 22 | -2.49 | 0.089 |
| | Amstelveen - Leiden | | -0.959 | 0.630 | 22 | -1.52 | 0.441 |
| | Amsterdam - Haarlem | | -0.003 | 0.545 | 22 | -0.01 | 1 |
| | Amsterdam - Leiden | | 0.506 | 0.589 | 22 | 0.86 | 0.826 |
| | Haarlem - Leiden | | 0.509 | 0.589 | 22 | 0.86 | 0.823 |
| | Amstelveen - Amsterdam | Urban | -0.120 | 0.545 | 22 | -0.22 | 0.996 |
| | Amstelveen - Haarlem | | -0.674 | 0.545 | 22 | -1.24 | 0.611 |
| | Amstelveen - Leiden | | -1.108 | 0.545 | 22 | -2.03 | 0.207 |
| | Amsterdam - Haarlem | | -0.554 | 0.545 | 22 | -1.02 | 0.742 |
| | Amsterdam - Leiden | | -0.988 | 0.545 | 22 | -1.8 | 0.295 |

| Trait | Comparison | Habitat | Estimate | SE | df | t / z | p |
|---|---|---|---|---|---|---|---|
| | Haarlem - Leiden | | -0.434 | 0.545 | 22 | -0.80 | 0.856 |
| Moisture preference | Amstelveen - Amsterdam | Rural | NA | NA | NA | -1.23 | 1 |
| | Amstelveen - Haarlem | | NA | NA | NA | -2.09 | 0.222 |
| | Amstelveen - Leiden | | NA | NA | NA | -0.20 | 1 |
| | Amsterdam - Haarlem | | NA | NA | NA | -0.93 | 1 |
| | Amsterdam - Leiden | | NA | NA | NA | 1.02 | 1 |
| | Haarlem - Leiden | | NA | NA | NA | 1.88 | 0.362 |
| | Amstelveen - Amsterdam | Urban | NA | NA | NA | 1.88 | 1 |
| | Amstelveen - Haarlem | | NA | NA | NA | 1.88 | 0.359 |
| | Amstelveen - Leiden | | NA | NA | NA | 0.49 | 1 |

| Trait | Comparison | Habitat | Estimate | SE | df | t / z | p |
|---|---|---|---|---|---|---|---|
| | Amsterdam - Haarlem | | NA | NA | NA | 1.64 | 0.610 |
| | Amsterdam - Leiden | | NA | NA | NA | 0.25 | 1 |
| | Haarlem - Leiden | | NA | NA | NA | -1.39 | 0.985 |
| Months active | Amstelveen - Amsterdam | Rural | -1.208 | 0.327 | 22 | -3.69 | **0.006** |
| | Amstelveen - Haarlem | | -1.639 | 0.327 | 22 | -5.01 | **0.000** |
| | Amstelveen - Leiden | | -1.829 | 0.350 | 22 | -5.23 | **0.000** |
| | Amsterdam - Haarlem | | -0.431 | 0.303 | 22 | -1.42 | 0.499 |
| | Amsterdam - Leiden | | -0.621 | 0.327 | 22 | -1.90 | 0.257 |
| | Haarlem - Leiden | | -0.190 | 0.327 | 22 | -0.58 | 0.937 |
| | Amstelveen - Amsterdam | Urban | -0.298 | 0.303 | 22 | -0.99 | 0.759 |
| | Amstelveen - Haarlem | | 0.548 | 0.303 | 22 | 1.81 | 0.295 |

| Trait | Comparison | Habitat | Estimate | SE | df | t / z | p |
|---|---|---|---|---|---|---|---|
| | Amstelveen - Leiden | | 0.525 | 0.303 | 22 | 1.73 | 0.331 |
| | Amsterdam - Haarlem | | 0.846 | 0.303 | 22 | 2.80 | **0.048** |
| | Amsterdam - Leiden | | 0.823 | 0.303 | 22 | 2.72 | 0.056 |
| | Haarlem - Leiden | | -0.023 | 0.303 | 22 | -0.08 | 1.000 |
| Nocturnality | Amstelveen - Amsterdam | Rural | -0.609 | 0.130 | 22 | -4.67 | 0.001 |
| | Amstelveen - Haarlem | | -0.545 | 0.130 | 22 | -4.18 | **0.002** |
| | Amstelveen - Leiden | | -0.223 | 0.139 | 22 | -1.60 | 0.401 |
| | Amsterdam - Haarlem | | 0.065 | 0.121 | 22 | 0.54 | 0.949 |
| | Amsterdam - Leiden | | 0.387 | 0.130 | 22 | 2.97 | **0.033** |
| | Haarlem - Leiden | | 0.322 | 0.130 | 22 | 2.47 | 0.093 |
| | Amstelveen - Amsterdam | Urban | 0.107 | 0.121 | 22 | 0.89 | 0.810 |

| Trait | Comparison | Habitat | Estimate | SE | df | t / z | p |
|---|---|---|---|---|---|---|---|
| | Amstelveen - Haarlem | | 0.043 | 0.121 | 22 | 0.36 | 0.984 |
| | Amstelveen - Leiden | | 0.195 | 0.121 | 22 | 1.61 | 0.392 |
| | Amsterdam - Haarlem | | -0.064 | 0.121 | 22 | -0.53 | 0.951 |
| | Amsterdam - Leiden | | 0.087 | 0.121 | 22 | 0.72 | 0.887 |
| | Haarlem - Leiden | | 0.151 | 0.121 | 22 | 1.25 | 0.601 |
| Overwintering stage | Amstelveen - Amsterdam | Rural | -0.525 | 0.205 | 22 | -2.56 | 0.077 |
| | Amstelveen - Haarlem | | -0.538 | 0.205 | 22 | -2.63 | 0.068 |
| | Amstelveen - Leiden | | -0.465 | 0.219 | 22 | -2.12 | 0.177 |
| | Amsterdam - Haarlem | | -0.013 | 0.190 | 22 | -0.07 | 1.000 |
| | Amsterdam - Leiden | | 0.060 | 0.205 | 22 | 0.29 | 0.991 |

| Trait | Comparison | Habitat | Estimate | SE | df | t / z | p |
|---|---|---|---|---|---|---|---|
| | Haarlem - Leiden | | 0.073 | 0.205 | 22 | 0.36 | 0.984 |
| | Amstelveen - Amsterdam | Urban | 0.054 | 0.190 | 22 | 0.28 | 0.992 |
| | Amstelveen - Haarlem | | -0.095 | 0.190 | 22 | -0.50 | 0.958 |
| | Amstelveen - Leiden | | 0.212 | 0.190 | 22 | 1.12 | 0.681 |
| | Amsterdam - Haarlem | | -0.149 | 0.190 | 22 | -0.78 | 0.861 |
| | Amsterdam - Leiden | | 0.159 | 0.190 | 22 | 0.84 | 0.836 |
| | Haarlem - Leiden | | 0.307 | 0.190 | 22 | 1.62 | 0.388 |
| Timing of reproduction | Amstelveen - Amsterdam | Rural | 1.749 | 0.720 | 22 | 2.43 | 0.101 |
| | Amstelveen - Haarlem | | 1.789 | 0.720 | 22 | 2.48 | 0.091 |
| | Amstelveen - Leiden | | 1.668 | 0.770 | 22 | 2.17 | 0.164 |

| Trait | Comparison | Habitat | Estimate | SE | df | t / z | p |
|---|---|---|---|---|---|---|---|
| | Amsterdam - Haarlem | | 0.039 | 0.667 | 22 | 0.06 | 1.000 |
| | Amsterdam - Leiden | | -0.081 | 0.720 | 22 | -0.11 | 0.999 |
| | Haarlem - Leiden | | -0.120 | 0.720 | 22 | -0.17 | 0.998 |
| | Amstelveen - Amsterdam | Urban | -0.323 | 0.667 | 22 | -0.48 | 0.962 |
| | Amstelveen - Haarlem | | 0.025 | 0.667 | 22 | 0.04 | 1.000 |
| | Amstelveen - Leiden | | -1.096 | 0.667 | 22 | -1.64 | 0.376 |
| | Amsterdam - Haarlem | | 0.348 | 0.667 | 22 | 0.52 | 0.953 |
| | Amsterdam - Leiden | | -0.773 | 0.667 | 22 | -1.16 | 0.658 |
| | Haarlem - Leiden | | -1.121 | 0.667 | 22 | -1.68 | 0.357 |

**Table S6A** Species are ordered from high to low total abundance across all samples excluding locations “In de Schuur” (Amstelveen - rural) and “h5.5” (Leiden - rural). Specimens were caught with pitfall traps in the urban habitat and rural habitat of four cities (Amstelveen, Amsterdam, Haarlem and Leiden). Within each habitat, four sites were combined to determine the total number of individuals per habitat for each species in each city

| **Species** | **Total** | **Amstelveen** | | **Amsterdam** | | **Haarlem** | | **Leiden** | |
|---|---|---|---|---|---|---|---|---|---|
| | | urban | rural | urban | rural | urban | rural | urban | rural |
| *Nebria brevicollis* | 1606 | 390 | 33 | 224 | 1 | 203 | 23 | 732 | 0 |
| *Anchomenus dorsalis* | 367 | 203 | 108 | 35 | 8 | 3 | 0 | 10 | 0 |
| *Poecilus versicolor* | 316 | 3 | 0 | 1 | 190 | 1 | 113 | 6 | 2 |
| *Leistus fulvibarbis* | 294 | 168 | 63 | 58 | 0 | 3 | 0 | 1 | 1 |
| *Pterostichus melanarius* | 235 | 40 | 0 | 16 | 2 | 14 | 1 | 161 | 1 |
| *Carabus auratus* | 187 | 0 | 187 | 0 | 0 | 0 | 0 | 0 | 0 |
| *Notiophilus rufipes* | 185 | 39 | 35 | 13 | 0 | 52 | 0 | 39 | 7 |
| *Badister lacertosus* | 147 | 17 | 28 | 19 | 5 | 18 | 0 | 15 | 45 |
| *Poecilus cupreus* | 141 | 1 | 0 | 1 | 9 | 0 | 130 | 0 | 0 |
| *Pterostichus vernalis* | 127 | 7 | 6 | 29 | 22 | 1 | 54 | 2 | 6 |
| *Pterostichus nigrita* | 120 | 0 | 0 | 0 | 2 | 0 | 118 | 0 | 0 |

| Species | Total | Amstelveen | | Amsterdam | | Haarlem | | Leiden | |
|---|---|---|---|---|---|---|---|---|---|
| | | urban | rural | urban | rural | urban | rural | urban | rural |
| *Harpalus tardus* | 118 | 1 | 0 | 1 | 57 | 42 | 0 | 11 | 6 |
| *Amara aenea* | 117 | 3 | 4 | 1 | 98 | 3 | 2 | 3 | 3 |
| *Harpalus anxius* | 92 | 0 | 0 | 0 | 81 | 11 | 0 | 0 | 0 |
| *Amara similata* | 87 | 0 | 2 | 0 | 60 | 1 | 1 | 11 | 12 |
| *Badister bullatus* | 86 | 2 | 9 | 51 | 1 | 10 | 0 | 2 | 11 |
| *Anisodactylus binotatus* | 85 | 1 | 4 | 10 | 53 | 1 | 8 | 7 | 1 |
| *Amara communis* | 83 | 0 | 3 | 1 | 48 | 4 | 13 | 3 | 11 |
| *Pterostichus strenuus* | 82 | 11 | 2 | 17 | 5 | 0 | 44 | 1 | 1 |
| *Amara lunicollis* | 75 | 0 | 2 | 0 | 56 | 0 | 8 | 0 | 9 |
| *Ophonus rufibarbis* | 49 | 3 | 40 | 0 | 2 | 2 | 0 | 0 | 2 |
| *Acupalpus exiguus* | 42 | 0 | 1 | 0 | 22 | 0 | 19 | 0 | 0 |
| *Carabus granulatus* | 38 | 0 | 1 | 0 | 11 | 0 | 26 | 0 | 0 |
| *Pterostichus anthracinus* | 38 | 2 | 1 | 0 | 16 | 0 | 19 | 0 | 0 |
| *Harpalus rufipes* | 30 | 0 | 2 | 0 | 4 | 13 | 0 | 11 | 0 |
| *Notiophilus biguttatus* | 28 | 8 | 5 | 3 | 0 | 5 | 0 | 4 | 3 |

| Species | Total | Amstelveen | | Amsterdam | | Haarlem | | Leiden | |
|---|---|---|---|---|---|---|---|---|---|
| | | urban | rural | urban | rural | urban | rural | urban | rural |
| *Clivina fossor* | 27 | 0 | 2 | 1 | 5 | 0 | 9 | 9 | 1 |
| *Bembidion guttula* | 26 | 0 | 0 | 0 | 0 | 0 | 25 | 1 | 0 |
| *Asaphidion curtum* | 24 | 1 | 0 | 1 | 0 | 0 | 0 | 20 | 2 |
| *Harpalus affinis* | 24 | 1 | 0 | 4 | 1 | 12 | 0 | 6 | 0 |
| *Oodes helopioides* | 23 | 0 | 0 | 0 | 2 | 0 | 21 | 0 | 0 |
| *Amara ovata* | 17 | 1 | 5 | 1 | 5 | 0 | 0 | 4 | 1 |
| *Syntomus foveatus* | 17 | 0 | 0 | 0 | 16 | 1 | 0 | 0 | 0 |
| *Amara plebeja* | 16 | 0 | 1 | 0 | 8 | 0 | 2 | 0 | 5 |
| *Bembidion properans* | 16 | 0 | 1 | 0 | 2 | 0 | 10 | 0 | 3 |
| *Leistus rufomarginatus* | 16 | 4 | 2 | 8 | 0 | 2 | 0 | 0 | 0 |
| *Pterostichus diligens* | 16 | 0 | 0 | 0 | 0 | 4 | 11 | 1 | 0 |
| *Agonum viduum* | 15 | 0 | 0 | 0 | 0 | 0 | 15 | 0 | 0 |
| *Agonum muelleri* | 14 | 1 | 0 | 0 | 13 | 0 | 0 | 0 | 0 |
| *Calathus rotundicollis* | 14 | 0 | 12 | 0 | 0 | 2 | 0 | 0 | 0 |
| *Loricera pilicornis* | 13 | 6 | 4 | 0 | 1 | 0 | 0 | 2 | 0 |

| Species | Total | Amstelveen | | Amsterdam | | Haarlem | | Leiden | |
|---|---|---|---|---|---|---|---|---|---|
| | | urban | rural | urban | rural | urban | rural | urban | rural |
| *Notiophilus substriatus* | 12 | 0 | 8 | 1 | 0 | 0 | 3 | 0 | 0 |
| *Paranchus albipes* | 12 | 12 | 0 | 0 | 0 | 0 | 0 | 0 | 0 |
| *Calathus melanocephalus* | 11 | 0 | 0 | 0 | 10 | 1 | 0 | 0 | 0 |
| *Stenolophus mixtus* | 11 | 1 | 2 | 0 | 5 | 0 | 3 | 0 | 0 |
| *Trechus obtusus* | 11 | 1 | 2 | 0 | 2 | 0 | 0 | 4 | 2 |
| *Bembidion lunulatum* | 10 | 0 | 0 | 0 | 0 | 0 | 9 | 0 | 1 |
| *Pterostichus minor* | 9 | 0 | 0 | 0 | 0 | 0 | 9 | 0 | 0 |
| *Harpalus luteicornis* | 8 | 0 | 0 | 0 | 7 | 0 | 0 | 0 | 1 |
| *Acupalpus dubius* | 7 | 0 | 0 | 0 | 7 | 0 | 0 | 0 | 0 |
| *Bembidion biguttatum* | 7 | 6 | 0 | 0 | 0 | 0 | 0 | 1 | 0 |
| *Stomis pumicatus* | 7 | 3 | 2 | 0 | 2 | 0 | 0 | 0 | 0 |
| *Asaphidion flavipes* | 6 | 1 | 1 | 2 | 0 | 1 | 0 | 1 | 0 |
| *Bembidion tetracolum* | 6 | 3 | 3 | 0 | 0 | 0 | 0 | 0 | 0 |
| *Demetrias atricapillus* | 6 | 0 | 3 | 0 | 0 | 0 | 3 | 0 | 0 |
| *Harpalus rubripes* | 6 | 0 | 0 | 0 | 6 | 0 | 0 | 0 | 0 |

| Species | Total | Amstelveen | | Amsterdam | | Haarlem | | Leiden | |
|---|---|---|---|---|---|---|---|---|---|
| | | urban | rural | urban | rural | urban | rural | urban | rural |
| *Amara curta* | 5 | 0 | 2 | 0 | 1 | 0 | 0 | 0 | 2 |
| *Carabus nemoralis* | 5 | 0 | 5 | 0 | 0 | 0 | 0 | 0 | 0 |
| *Leistus ferrugineus* | 5 | 0 | 4 | 0 | 0 | 0 | 0 | 0 | 1 |
| *Amara anthobia* | 4 | 0 | 0 | 0 | 3 | 1 | 0 | 0 | 0 |
| *Amara familiaris* | 4 | 0 | 0 | 1 | 2 | 0 | 0 | 0 | 1 |
| *Badister sodalis* | 4 | 0 | 0 | 2 | 0 | 0 | 0 | 1 | 1 |
| *Bembidion lampros* | 4 | 3 | 0 | 0 | 0 | 0 | 0 | 1 | 0 |
| *Trechus quadristriatus* | 4 | 0 | 4 | 0 | 0 | 0 | 0 | 0 | 0 |
| *Bradycellus harpalinus* | 3 | 0 | 0 | 0 | 2 | 0 | 1 | 0 | 0 |
| *Amara fulva* | 2 | 0 | 0 | 0 | 0 | 0 | 0 | 2 | 0 |
| *Amara lucida* | 2 | 0 | 0 | 0 | 2 | 0 | 0 | 0 | 0 |
| *Bembidion obtusum* | 2 | 1 | 0 | 0 | 0 | 0 | 0 | 1 | 0 |
| *Dyschirius globosus* | 2 | 0 | 0 | 0 | 1 | 0 | 1 | 0 | 0 |
| *Harpalus latus* | 2 | 0 | 0 | 1 | 1 | 0 | 0 | 0 | 0 |
| *Notiophilus palustris* | 2 | 0 | 2 | 0 | 0 | 0 | 0 | 0 | 0 |

| Species | Total | Amstelveen | | Amsterdam | | Haarlem | | Leiden | |
|---|---|---|---|---|---|---|---|---|---|
| | | urban | rural | urban | rural | urban | rural | urban | rural |
| *Panagaeus bipustulatus* | 2 | 0 | 2 | 0 | 0 | 0 | 0 | 0 | 0 |
| *Stenolophus teutonus* | 2 | 0 | 0 | 0 | 0 | 0 | 2 | 0 | 0 |
| *Trechoblemus micros* | 2 | 1 | 0 | 1 | 0 | 0 | 0 | 0 | 0 |
| *Acupalpus parvulus* | 1 | 0 | 0 | 0 | 0 | 0 | 1 | 0 | 0 |
| *Amara bifrons* | 1 | 0 | 0 | 0 | 1 | 0 | 0 | 0 | 0 |
| *Amara convexior* | 1 | 0 | 1 | 0 | 0 | 0 | 0 | 0 | 0 |
| *Amara spreta* | 1 | 0 | 0 | 0 | 1 | 0 | 0 | 0 | 0 |
| *Badister unipustulatus* | 1 | 0 | 1 | 0 | 0 | 0 | 0 | 0 | 0 |
| *Bembidion quadrimaculatum* | 1 | 0 | 0 | 0 | 0 | 1 | 0 | 0 | 0 |
| *Bradycellus verbasci* | 1 | 1 | 0 | 0 | 0 | 0 | 0 | 0 | 0 |
| *Calathus cinctus* | 1 | 0 | 0 | 0 | 1 | 0 | 0 | 0 | 0 |
| *Clivina collaris* | 1 | 1 | 0 | 0 | 0 | 0 | 0 | 0 | 0 |
| *Oxypselaphus obscurus* | 1 | 0 | 0 | 0 | 0 | 0 | 0 | 0 | 1 |

| Species | Total | Amstelveen | | Amsterdam | | Haarlem | | Leiden | |
|---|---|---|---|---|---|---|---|---|---|
| | | urban | rural | urban | rural | urban | rural | urban | rural |
| *Pterostichus oblongopunctatus* | 1 | 0 | 1 | 0 | 0 | 0 | 0 | 0 | 0 |
| Total | 5249 | 947 | 607 | 503 | 860 | 412 | 704 | 1073 | 143 |

**Table S6B** Species are ordered from high to low total abundance across all samples (i.e. including samples "In de Schuur" (Amstelveen - rural) and "h5.5" (Leiden - rural) that had a too low sample coverage for our analyses). Specimens were caught with pitfall traps in the urban habitat and rural habitat of four cities (Amstelveen, Amsterdam, Haarlem and Leiden). Within each habitat, four sites, each with 5 pitfall traps, were combined over 10 weeks of sampling were collected, to determine the total number of individuals per habitat for each species in each city

| Species | Total | Amstelveen | | Amsterdam | | Haarlem | | Leiden | |
|---|---|---|---|---|---|---|---|---|---|
| | | urban | rural | urban | rural | urban | rural | urban | rural |
| *Nebria brevicollis* | 1607 | 390 | 34 | 224 | 1 | 203 | 23 | 732 | 0 |
| *Anchomenus dorsalis* | 367 | 203 | 108 | 35 | 8 | 3 | 0 | 10 | 0 |
| *Poecilus versicolor* | 317 | 3 | 0 | 1 | 190 | 1 | 113 | 6 | 3 |
| *Leistus fulvibarbis* | 296 | 168 | 65 | 58 | 0 | 3 | 0 | 1 | 1 |
| *Pterostichus melanarius* | 235 | 40 | 0 | 16 | 2 | 14 | 1 | 161 | 1 |

| Species | Total | Amstelveen | | Amsterdam | | Haarlem | | Leiden | |
|---|---|---|---|---|---|---|---|---|---|
| | | urban | rural | urban | rural | urban | rural | urban | rural |
| *Carabus auratus* | 187 | 0 | 187 | 0 | 0 | 0 | 0 | 0 | 0 |
| *Notiophilus rufipes* | 185 | 39 | 35 | 13 | 0 | 52 | 0 | 39 | 7 |
| *Badister lacertosus* | 162 | 17 | 29 | 19 | 5 | 18 | 0 | 15 | 59 |
| *Poecilus cupreus* | 141 | 1 | 0 | 1 | 9 | 0 | 130 | 0 | 0 |
| *Pterostichus vernalis* | 128 | 7 | 6 | 29 | 22 | 1 | 54 | 2 | 7 |
| *Pterostichus nigrita* | 120 | 0 | 0 | 0 | 2 | 0 | 118 | 0 | 0 |
| *Harpalus tardus* | 118 | 1 | 0 | 1 | 57 | 42 | 0 | 11 | 6 |
| *Amara aenea* | 117 | 3 | 4 | 1 | 98 | 3 | 2 | 3 | 3 |
| *Harpalus anxius* | 92 | 0 | 0 | 0 | 81 | 11 | 0 | 0 | 0 |
| *Amara similata* | 88 | 0 | 2 | 0 | 60 | 1 | 1 | 11 | 13 |
| *Anisodactylus binotatus* | 86 | 1 | 4 | 10 | 53 | 1 | 8 | 7 | 2 |
| *Badister bullatus* | 86 | 2 | 9 | 51 | 1 | 10 | 0 | 2 | 11 |
| *Pterostichus strenuus* | 84 | 11 | 4 | 17 | 5 | 0 | 44 | 1 | 2 |
| *Amara communis* | 83 | 0 | 3 | 1 | 48 | 4 | 13 | 3 | 11 |
| *Amara lunicollis* | 75 | 0 | 2 | 0 | 56 | 0 | 8 | 0 | 9 |

| Species | Total | Amstelveen | | Amsterdam | | Haarlem | | Leiden | |
|---|---|---|---|---|---|---|---|---|---|
| | | urban | rural | urban | rural | urban | rural | urban | rural |
| *Ophonus rufibarbis* | 49 | 3 | 40 | 0 | 2 | 2 | 0 | 0 | 2 |
| *Acupalpus exiguus* | 42 | 0 | 1 | 0 | 22 | 0 | 19 | 0 | 0 |
| *Pterostichus anthracinus* | 40 | 2 | 2 | 0 | 16 | 0 | 19 | 0 | 1 |
| *Carabus granulatus* | 38 | 0 | 1 | 0 | 11 | 0 | 26 | 0 | 0 |
| *Harpalus rufipes* | 30 | 0 | 2 | 0 | 4 | 13 | 0 | 11 | 0 |
| *Clivina fossor* | 28 | 0 | 2 | 1 | 5 | 0 | 9 | 9 | 2 |
| *Notiophilus biguttatus* | 28 | 8 | 5 | 3 | 0 | 5 | 0 | 4 | 3 |
| *Bembidion guttula* | 26 | 0 | 0 | 0 | 0 | 0 | 25 | 1 | 0 |
| *Asaphidion curtum* | 24 | 1 | 0 | 1 | 0 | 0 | 0 | 20 | 2 |
| *Harpalus affinis* | 24 | 1 | 0 | 4 | 1 | 12 | 0 | 6 | 0 |
| *Oodes helopioides* | 23 | 0 | 0 | 0 | 2 | 0 | 21 | 0 | 0 |
| *Amara ovata* | 17 | 1 | 5 | 1 | 5 | 0 | 0 | 4 | 1 |
| *Syntomus foveatus* | 17 | 0 | 0 | 0 | 16 | 1 | 0 | 0 | 0 |
| *Amara plebeja* | 16 | 0 | 1 | 0 | 8 | 0 | 2 | 0 | 5 |
| *Bembidion properans* | 16 | 0 | 1 | 0 | 2 | 0 | 10 | 0 | 3 |

| Species | Total | Amstelveen | | Amsterdam | | Haarlem | | Leiden | |
|---|---|---|---|---|---|---|---|---|---|
| | | urban | rural | urban | rural | urban | rural | urban | rural |
| *Leistus rufomarginatus* | 16 | 4 | 2 | 8 | 0 | 2 | 0 | 0 | 0 |
| *Pterostichus diligens* | 16 | 0 | 0 | 0 | 0 | 4 | 11 | 1 | 0 |
| *Agonum viduum* | 15 | 0 | 0 | 0 | 0 | 0 | 15 | 0 | 0 |
| *Agonum muelleri* | 14 | 1 | 0 | 0 | 13 | 0 | 0 | 0 | 0 |
| *Calathus rotundicollis* | 14 | 0 | 12 | 0 | 0 | 2 | 0 | 0 | 0 |
| *Loricera pilicornis* | 13 | 6 | 4 | 0 | 1 | 0 | 0 | 2 | 0 |
| *Notiophilus substriatus* | 12 | 0 | 8 | 1 | 0 | 0 | 3 | 0 | 0 |
| *Paranchus albipes* | 12 | 12 | 0 | 0 | 0 | 0 | 0 | 0 | 0 |
| *Calathus melanocephalus* | 11 | 0 | 0 | 0 | 10 | 1 | 0 | 0 | 0 |
| *Stenolophus mixtus* | 11 | 1 | 2 | 0 | 5 | 0 | 3 | 0 | 0 |
| *Trechus obtusus* | 11 | 1 | 2 | 0 | 2 | 0 | 0 | 4 | 2 |
| *Bembidion lunulatum* | 10 | 0 | 0 | 0 | 0 | 0 | 9 | 0 | 1 |
| *Pterostichus minor* | 9 | 0 | 0 | 0 | 0 | 0 | 9 | 0 | 0 |
| *Harpalus luteicornis* | 8 | 0 | 0 | 0 | 7 | 0 | 0 | 0 | 1 |
| *Acupalpus dubius* | 7 | 0 | 0 | 0 | 7 | 0 | 0 | 0 | 0 |

| Species | Total | Amstelveen | | Amsterdam | | Haarlem | | Leiden | |
|---|---|---|---|---|---|---|---|---|---|
| | | urban | rural | urban | rural | urban | rural | urban | rural |
| *Bembidion biguttatum* | 7 | 6 | 0 | 0 | 0 | 0 | 0 | 1 | 0 |
| *Stomis pumicatus* | 7 | 3 | 2 | 0 | 2 | 0 | 0 | 0 | 0 |
| *Asaphidion flavipes* | 6 | 1 | 1 | 2 | 0 | 1 | 0 | 1 | 0 |
| *Bembidion tetracolum* | 6 | 3 | 3 | 0 | 0 | 0 | 0 | 0 | 0 |
| *Demetrias atricapillus* | 6 | 0 | 3 | 0 | 0 | 0 | 3 | 0 | 0 |
| *Harpalus rubripes* | 6 | 0 | 0 | 0 | 6 | 0 | 0 | 0 | 0 |
| *Amara curta* | 5 | 0 | 2 | 0 | 1 | 0 | 0 | 0 | 2 |
| *Badister sodalis* | 5 | 0 | 0 | 2 | 0 | 0 | 0 | 1 | 2 |
| *Bembidion lampros* | 5 | 3 | 0 | 0 | 0 | 0 | 0 | 1 | 1 |
| *Carabus nemoralis* | 5 | 0 | 5 | 0 | 0 | 0 | 0 | 0 | 0 |
| *Leistus ferrugineus* | 5 | 0 | 4 | 0 | 0 | 0 | 0 | 0 | 1 |
| *Amara anthobia* | 4 | 0 | 0 | 0 | 3 | 1 | 0 | 0 | 0 |
| *Amara familiaris* | 4 | 0 | 0 | 1 | 2 | 0 | 0 | 0 | 1 |
| *Panagaeus bipustulatus* | 4 | 0 | 4 | 0 | 0 | 0 | 0 | 0 | 0 |
| *Trechus quadristriatus* | 4 | 0 | 4 | 0 | 0 | 0 | 0 | 0 | 0 |

| Species | Total | Amstelveen | | Amsterdam | | Haarlem | | Leiden | |
|---|---|---|---|---|---|---|---|---|---|
| | | urban | rural | urban | rural | urban | rural | urban | rural |
| *Bradycellus harpalinus* | 3 | 0 | 0 | 0 | 2 | 0 | 1 | 0 | 0 |
| *Amara fulva* | 2 | 0 | 0 | 0 | 0 | 0 | 0 | 2 | 0 |
| *Amara lucida* | 2 | 0 | 0 | 0 | 2 | 0 | 0 | 0 | 0 |
| *Bembidion obtusum* | 2 | 1 | 0 | 0 | 0 | 0 | 0 | 1 | 0 |
| *Bembidion quadrimaculatum* | 2 | 0 | 0 | 0 | 0 | 1 | 0 | 0 | 1 |
| *Dyschirius globosus* | 2 | 0 | 0 | 0 | 1 | 0 | 1 | 0 | 0 |
| *Harpalus latus* | 2 | 0 | 0 | 1 | 1 | 0 | 0 | 0 | 0 |
| *Notiophilus palustris* | 2 | 0 | 2 | 0 | 0 | 0 | 0 | 0 | 0 |
| *Stenolophus teutonus* | 2 | 0 | 0 | 0 | 0 | 0 | 2 | 0 | 0 |
| *Trechoblemus micros* | 2 | 1 | 0 | 1 | 0 | 0 | 0 | 0 | 0 |
| *Acupalpus parvulus* | 1 | 0 | 0 | 0 | 0 | 0 | 1 | 0 | 0 |
| *Amara bifrons* | 1 | 0 | 0 | 0 | 1 | 0 | 0 | 0 | 0 |
| *Amara convexior* | 1 | 0 | 1 | 0 | 0 | 0 | 0 | 0 | 0 |
| *Amara spreta* | 1 | 0 | 0 | 0 | 1 | 0 | 0 | 0 | 0 |

| Species | Total | Amstelveen | | Amsterdam | | Haarlem | | Leiden | |
|---|---|---|---|---|---|---|---|---|---|
| | | urban | rural | urban | rural | urban | rural | urban | rural |
| *Badister unipustulatus* | 1 | 0 | 1 | 0 | 0 | 0 | 0 | 0 | 0 |
| *Bradycellus verbasci* | 1 | 1 | 0 | 0 | 0 | 0 | 0 | 0 | 0 |
| *Calathus cinctus* | 1 | 0 | 0 | 0 | 1 | 0 | 0 | 0 | 0 |
| *Clivina collaris* | 1 | 1 | 0 | 0 | 0 | 0 | 0 | 0 | 0 |
| *Oxypselaphus obscurus* | 1 | 0 | 0 | 0 | 0 | 0 | 0 | 0 | 1 |
| *Pterostichus oblongopunctatus* | 1 | 0 | 1 | 0 | 0 | 0 | 0 | 0 | 0 |
| Total | 5281 | 947 | 615 | 503 | 860 | 412 | 704 | 1073 | 167 |

**References**

Boeken, M., Desender, K., Drost, B., Van Gijzen, T., Koese, B., Muilwijk, J., Turin, H. and Vermeulen, R. 2002. De loopkevers van Nederland en Vlaanderen. - Jeugdbondsuitgeverij.

Buuren, S. van and Groothuis-Oudshoorn, K. 2011. mice: multivariate imputation by chained equations in R. - Journal of Statistical Software 45: 1–67.

Hsieh, T. C., Ma, K. H. and Chao, A. 2016. iNEXT: an R package for rarefaction and extrapolation of species diversity (Hill numbers). - Methods in Ecology and Evolution 7: 1451–1456.

Muilwijk, J., Felix, R., Dekoninck, W. and Bleich, O. 2015. De loopkevers van Nederland en België (Carabidae). - Nederlandse Entomologische Vereniging, Naturalis Biodiversity Center, EIS Kenniscentrum Insecten.

Turin, H. 2000. De Nederlandse loopkevers, verspreiding en oecologie (Coleoptera: Carabidae). - Nationaal Natuurhistorisch Museum Naturalis, KNNV Uitgeverij, EIS-Nederland, Leiden.
